# Inhibition of Chk1 by miR-320c increases oxaliplatin responsiveness in triple-negative breast cancer

**DOI:** 10.1038/s41389-020-00275-x

**Published:** 2020-10-11

**Authors:** Sera Lim, Yesol Kim, Soo-Been Lee, Hyeok-Gu Kang, Da-Hyun Kim, Jee Won Park, Daeun Chung, Hyunkyung Kong, Kyung Hyun Yoo, Yonghwan Kim, Wonshik Han, Kyung-Hee Chun, Jong Hoon Park

**Affiliations:** 1 Department of Biological Sciences, Sookmyung Women’s University, Seoul, Republic of Korea; 2 Department of Biochemistry and Molecular Biology, Yonsei University College of Medicine, Seoul, Republic of Korea; 3 Cancer Research Institute, Seoul National University College of Medicine, Seoul, Republic of Korea; 4 Department of Surgery, Seoul National University College of Medicine, Seoul, Republic of Korea

**Keywords:** Breast cancer, Non-coding RNAs, DNA damage response

## Abstract

Checkpoint kinase 1 (Chk1) expression is enhanced in most cancers owing to oncogenic activation and constant replicative stress. Chk1 inactivation is a promising cancer therapy, as its inactivation leads to genomic instability, chromosomal catastrophe, and cancer cell death. Herein, we observed that miR-320c, downregulated in triple-negative breast cancer (TNBC) patients, can target Chk1. In addition, downregulated miR-320c expression was associated with poor overall survival in TNBC patients. As Chk1 was associated with the DNA damage response (DDR), we investigated the effect of miR-320c on DDR in TNBC cells. To induce DNA damage, we used platinum-based drugs, especially oxaliplatin, which is most effective with miR-320c. We observed that overexpression of miR-320c in TNBC regulated the oxaliplatin responsiveness by mediating DNA damage repair through the negative regulation of Chk1 in vitro. Furthermore, using a xenograft model, a combination of miR-320c mimic and oxaliplatin effectively inhibited tumor progression. These investigations indicate the potential of miR-320c as a marker of oxaliplatin responsiveness and a therapeutic target to increase the efficacy of chemotherapy in TNBC.

## Introduction

Breast cancer is the most common cancers in women worldwide, and triple-negative breast cancer (TNBC), one of its most aggressive forms, accounts for 12–20% of all breast cancer cases. TNBC is characterized by the absence of hormonal membrane receptors, estrogen receptor (ER), progesterone receptor (PR), and human epidermal growth factor receptor (HER2)^1^. TNBC has the worst prognosis among all breast cancer subtypes^2^ and, unlike other breast cancer subtypes (Luminal A, Luminal B, and HER2), the lack of these three receptors makes its treatment especially challenging, as these receptors normally serve as therapeutic targets^3^. Therefore, various studies have been performed to find biomarkers for predicting treatment results and for use as new therapeutic targets^4^.

MicroRNAs (miRNAs) are small noncoding RNAs which regulate gene expression by binding to the 3′-untranslated region (UTR) of target mRNAs^5^. Numerous studies have described the importance of miRNAs in biological processes, including cell proliferation, apoptosis, signal transduction; therefore, they are also related to cancer^6^. In addition, miRNAs may play a role in drug responsiveness and resistance by altering target gene expression in breast cancer^7^. Previous studies have reported that the miR-320 family is related to various types of cancer, such as ovarian and gastric cancer, and that it is involved in reprogramming of the tumor microenvironment^8–10^. Moreover, miR-320c has been associated with gemcitabine resistance in pancreatic cancer via SMARCC1 regulation^11^, and oncogenic behavior in bladder cancer by regulating CDK6 expression^12^.

In general, the survival of cancer cells relies on buffering the consequences of increased stress levels, such as DNA damage and replication stress, induced during the tumorigenic process. Therefore, to inhibit cancer cells specifically, it is required to inhibit stress‐reducing pathways and induce extra stress to critical levels^13^. Chemotherapy drugs that target DNA repair (platinum compounds) or p53 (taxanes) are used for TNBC patients in many cases^1^. As for the platinum compounds, the clinical activity of cisplatin and carboplatin against breast cancer has been reported several times, but their clinical activity against TNBC tumors has been limited^14^. Therefore, a new strategy for TNBC patients is needed. Oxaliplatin, a third-generation diaminocyclohexane (DACH) platinum compound, disrupts DNA replication and transcription by forming intrastrand DNA adducts. Several platinum analogs are available to overcome cisplatin and carboplatin resistance. As revealed in numerous preclinical evaluations and early clinical trial, oxaliplatin is reportedly the most effective compound in cisplatin and carboplatin-resistant cells, distinct from cisplatin in DNA binding, adduct formation, and apoptosis^15^. In a previous study, oxaliplatin showed antitumor activity in ovarian cancer, non-small-cell lung cancer, and breast cancer. However, given the heterogeneity of TNBC, it is still difficult to predict the prognosis and establish therapeutic strategies for TNBC.

DNA damage activates DNA damage response (DDR) by signaling pathways mediated by Rad9-Hus1-Rad1 (9-1-1)-ATR-Chk1, resulting in the activation of DNA repair pathways, like homologous recombination (HR). Thus, activated Chk1 allows to limit chemotherapeutic and radiotherapeutic efficacy^16,17^. Indeed, numerous studies attempt to identify Chk1 inhibitors to enhance the effect of chemotherapy^17^. Besides, it was reported that there are several miRNAs known to regulate Chk1 in non-small cell lung cancer^18^. However, there is no study about the effect of miRNA on the responsiveness to oxaliplatin in TNBC by regulating Chk1. In this study, we demonstrate that miR-320c may serve as a prognostic marker of oxaliplatin by regulating DDR through the expression of Chk1.

## Results

### The expression of microRNA-320c is dysregulated in TNBC

**Fig. 1:**
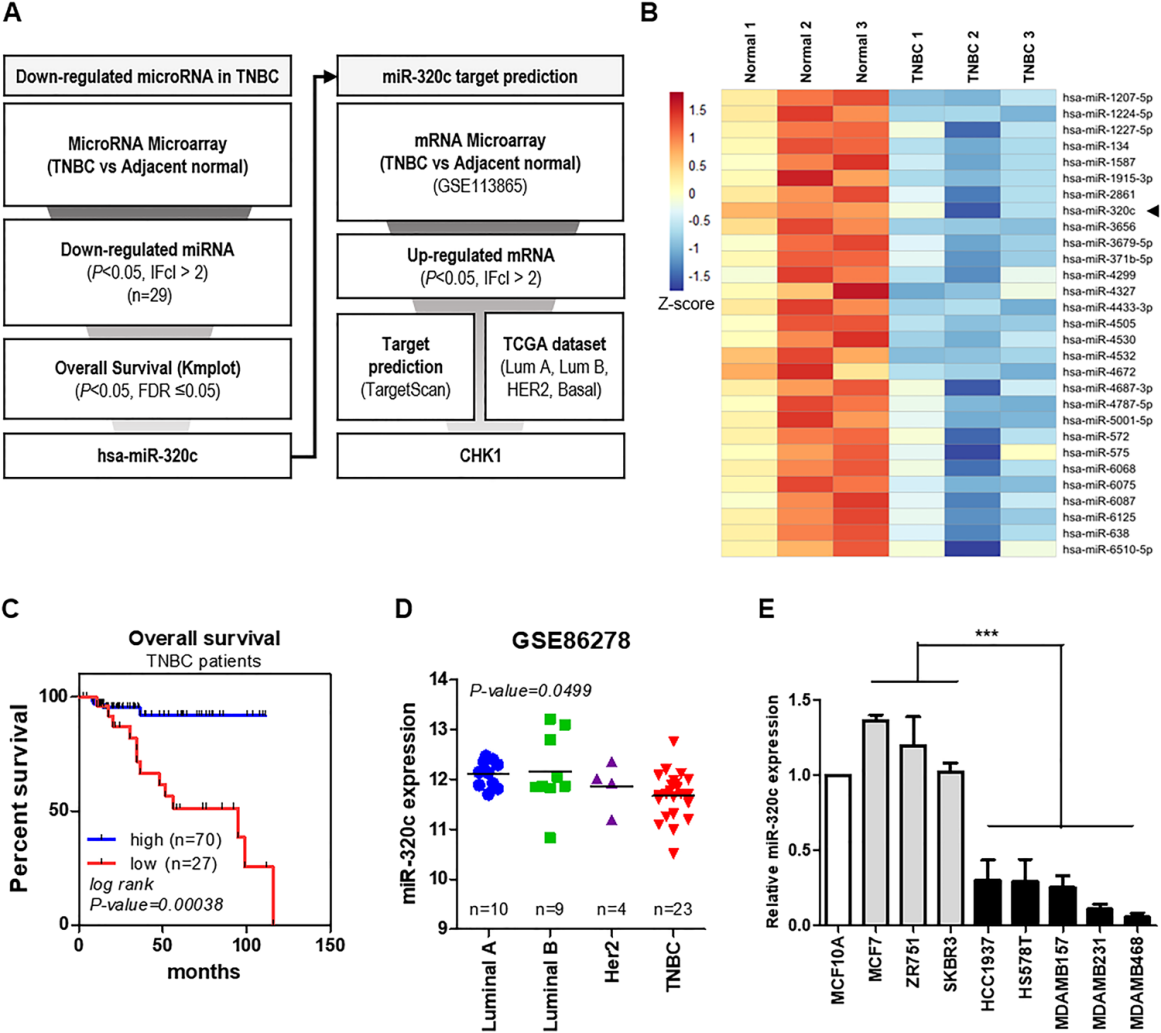
miR-320c expression is downregulated in triple-negative breast cancer (TNBC). **A** The scheme of this study is shown. **B** The heatmap of miRNAs with lower expression in the TNBC patients’ tumor tissues (*n* = 3) compared to normal adjacent tissues (*n* = 3). Expression levels (*Z* score) are represented by colored bars; higher (red) or lower (blue). **C** Kaplan–Meier analysis of overall survival of TNBC patients (*n* = 97) from the KM plotter database (www.kmplot.com)^20,52^. *P* value was calculated with the log-rank test. Patients were stratified into low- (red) (*n* = 70) and high- (blue) (*n* = 27) miR-320c expression based on autoselect best cutoff. **D** GEO dataset analysis showing the miR-320c differential expression in Luminal A (*n* = 10), Luminal B (*n* = 9), HER2 (*n* = 4), and TNBC (*n* = 23). *P* value was calculated using one-way ANOVA. The hsa-miR-320c_st (probe) expression data were used for analysis. **E** miR-320c expression level in breast cancer cell lines by using TaqMan quantitative RT-PCR. RNU48 was used for endogenous control. The data are representative of three independent experiments. ***P < 0.001.

We aimed to discover miRNA regulation in TNBC by integrated analysis from previous miRNA microarray and mRNA microarray data^19^ in TNBC tissues and adjacent normal tissues. In Fig. 1A, the flow chart describes the analysis performed to identify miRNAs and their target gene. First, we selected significant miRNAs (*n* = 29) with an absolute value of at least 2 (|fc | > 2), and a *P* value < 0.05 in the microRNA microarray (Fig. 1B and Supplementary Table 1). Next, the Kaplan–Meier analysis was performed using data from TNBC patients in TCGA (*n* = 97)^20^. Using overall survival analysis, we eliminated miRNAs that failed to precisely match TCGA miRNA ID and our miRNA microarray data (*n* = 23). Among the selected miRNAs, only hsa-miR-320c (miR-320c) expression revealed a significant association with the poor overall survival rate observed in the TNBC patient group (*P* value = 0.00038, FDR = 0.05); however, other miRNAs (miR-4505, miR-4672, and miR-6087) were failed to demonstrate statistical significance (Fig. 1C and Supplementary Fig. 1). Furthermore, miR-320c expression was decreased by approximately twofold in TNBC patient microRNA microarray data (*P* value = 0.043).

Interestingly, a significant association was noted between poor overall survival (OS) rates and low miR-320c expression in the TNBC patient group, but not in the non-TNBC group (Luminal A, Luminal B, Her2) (Supplementary Fig. 2A). Furthermore, the GEO dataset analysis^21^ revealed that the expression of miR-320c was decreased in TNBC subtypes compared to that in other non-TNBC subtypes (*P* value = 0.0499) (Fig. 1D). Consistently, we observed that miR-320c expression reduced in TNBC not only with respect to the normal breast cancer cell line MCF-10A but also in comparison to the non-TNBC breast cancer cell lines (Fig. 1E). Further, through a Kaplan–Meier analysis using TCGA pan-cancer atlas data^22^, we found five cancer types, which showed a significant association between lower miR-320c expression and poor OS; this included breast cancer (Supplementary Fig. 2B). These results collectively indicate that miR-320c was downregulated in TNBC cells and can be a potential marker in cancer.

### Chk1 is directly targeted by miR-320c

**Fig. 2:**
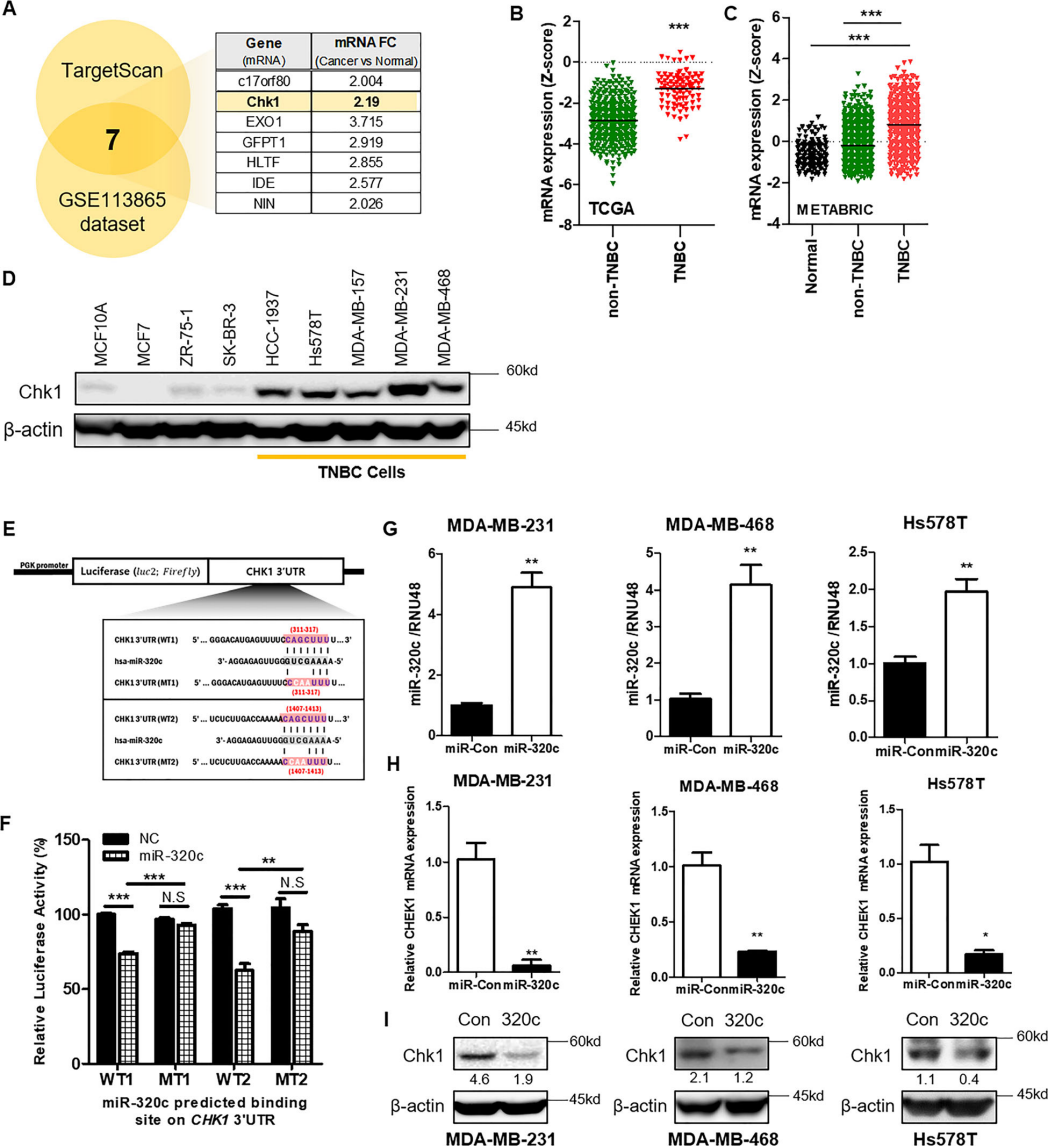
Chk1 expression is negatively regulated by miR-320c binding. **A** Table showing the miR-320c potential target that had negatively correlated expression patterns with miR-320c in triple-negative breast cancer (TNBC) tumor tissues and predicted to have miR-320c-binding sites by using TargetScan. **B** The expression level of Chk1 mRNA across different subtypes of breast cancer using TCGA datasets from cBioPortal^53,54^. Non-TNBC included Luminal A, Luminal B, and HER2 subtypes. TNBC included claudin-low and basal-like subtype. **C** The expression level of Chk1 was analyzed using METABRIC datasets from cBioPortal. Non-TNBC subtypes: Luminal A, Luminal B, and HER2. TNBC subtypes: claudin-low and basal-like. **D** Western blot analysis of Chk1 in breast cancer cell lines. β-actin was used as the loading control. **E** The sequence alignment of miR-320c and the 3′-UTR of CHK1 containing two binding sites. WT1 and 2; wild-type CHK1 3′-UTR seed sequence, MT1 and 2; mutant CHK1 3′-UTR seed sequence. The mutated sequences are shown in white letters. **F** Luciferase activity of the wild-type (WT) or mutant 3′-UTR construct (MT) of CHK1 in HEK293T cells after the transfection with miR-320c mimic (miR-320c) or negative control mimic (NC). N.S. not significant. **G** qRT-PCR analysis showing miR-320c expression after transfection with empty vector (miR-con) and miR-320c vector (miR-320c) in TNBC cell lines (MDA-MB-231, MDA-MB-468, and Hs578T) for 48 h. **H**, **I** Chk1 expression (mRNA and protein) when transfected with miR-con and miR-320c in TNBC cells (MDA-MB-231, MDA-MB-468, and Hs578T). The quantification of Chk1 expression in western blot was normalized by β-actin, used as the loading control. The data of **D**, **F**–**H**, and **I** are representative of three independent experiments. *P < 0.05, **P < 0.0.01, ***P < 0.001.

As described in Fig. 1A, to find the target genes of miR-320c, an integrated analysis of mRNA microarray data of a previous study was performed^19^. To evaluate target genes of miR-320c, we selected significant mRNAs (*n* = 1008) with an absolute value of at least 2 (|fc | =2), and *P* value<0.05 in mRNA microarray. Next, 847 candidate genes containing conserved miR-320c-binding sequences were discovered using TargetScan (http://www.targetscan.org)^23^. From these, we selected seven candidate genes that had a significant negative correlation with miR-320c with respect to the expression levels (Fig. 2A). To further confirm these results and using a TCGA dataset^24^, we found that Chk1 expression was increased in TNBC (*P* < 0.0001), but six genes were not significant compared to TNBC and other subtypes (Fig. 2B and Supplementary Fig. 3A). Additionally, Chk1 was upregulated in TNBC compared to non-TNBC in METABRIC dataset^25^ (Fig. 2C).

Chk1 was also included in among the 58 gene candidates predicted to be targets of miR-320c, in four databases (Supplementary Fig. S4): TargetScan^23^, miRwalk 3.0^26^, DIANA-microT web server v5.0^27,28^ and miRDB^29,30^. Chk1 expression showed an overall increased pattern in TNBC cell lines and GSE cell lines data^31^ (Fig. 2D and Supplementary Fig. S3B). Furthermore, Chk1 mRNA expression analysis was performed using TCGA pan-cancer data^32^. Based on this analysis, Chk1 mRNA levels were overall upregulated in tumors compared to normal in cancer type with poor OS associated with low miR-320c expression (Supplementary Fig. 3C).

Next, we investigated whether miR-320c regulated Chk1 expression in TNBC cell lines. Toward this, we performed a dual-luciferase reporter assay using two constructs of CHK1 with mutant 3′-UTRs, based on the fact that microRNAs target 3′-UTR regions^33^. In Fig. 2E, the miR-320c sequence and each complementary seed sequence region of miR-320c in CHK1 3′-UTR is illustrated. The luciferase activities of the wild-type CHK1 3′-UTR constructs were highly attenuated up on miR-320c overexpression. However, both of the mutant CHK1 3′-UTR constructs did not show inhibition of luciferase activities (Fig. 2F). Next, when miR-320c was transiently overexpressed (Fig. 2G), both mRNA and protein expression of Chk1 were suppressed in all three TNBC cell lines (Fig. 2H, I). Collectively, we demonstrated that Chk1 expression is negatively correlated with miR-320c expression in the TNBC tissue and TNBC cell lines, and miR-320c regulates Chk1.

### Upregulation of miR-320c increases drug responsiveness by modulating Chk1 in TNBC cells, in vitro

**Fig. 3:**
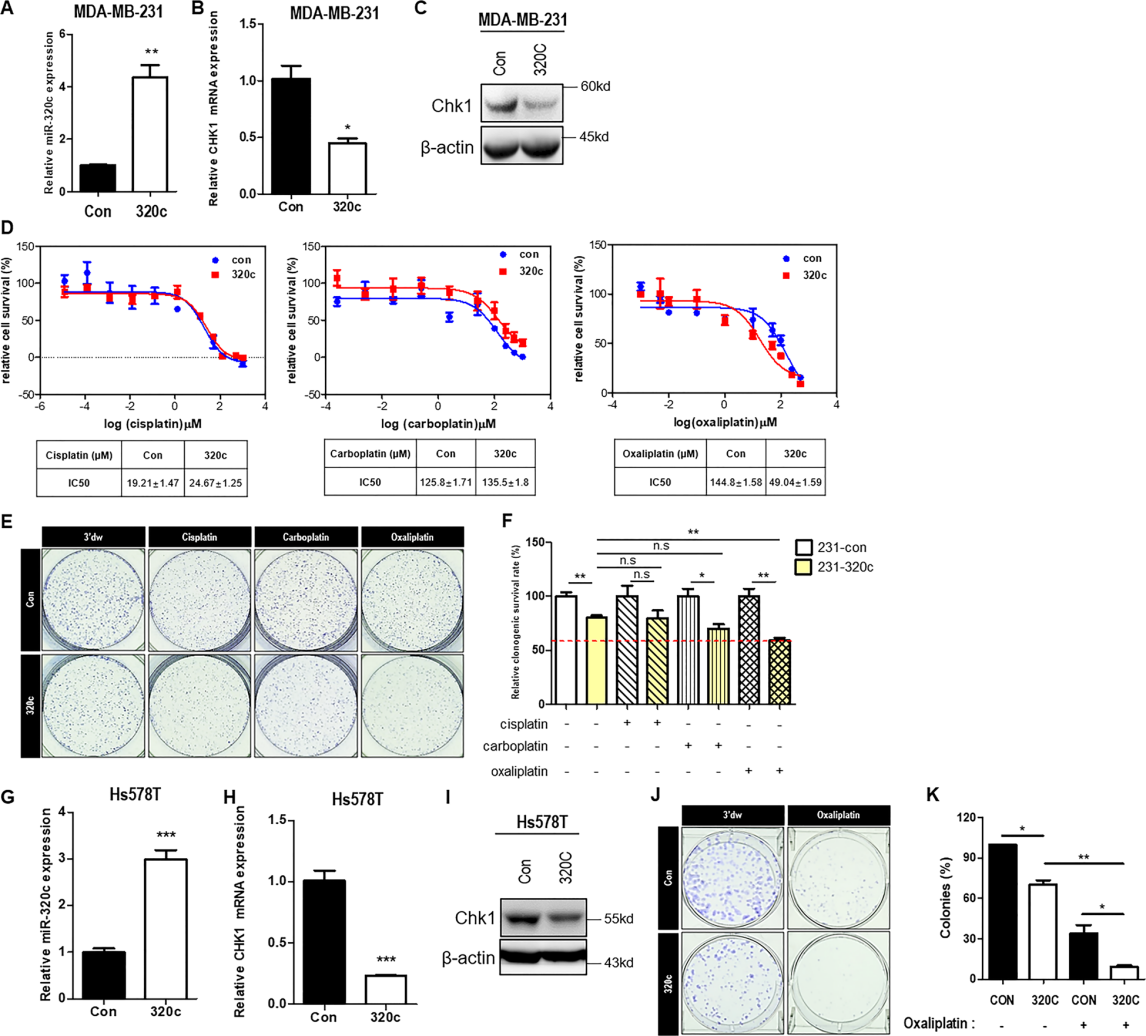
Upregulated miR-320c inhibits clonogenic survival by regulating Chk1, in vitro. **A** miR-320c expression level in MDA-MB-231 stable cell lines determined using TaqMan quantitative RT-PCR. Con; control miRNA overexpressing MDA-MB-231 stable cell line, 320c; miR-320c overexpressing MDA-MB-231 stable cell line. RNU48 was used for endogenous control. **B** CHK1 mRNA levels in MDA-MB-231 stable cells (Con and 320c). 18s rRNA was used as the endogenous control. **C** Western blot analysis of Chk1 in MDA-MB-231 stable cells (Con, 320c). β-actin was used as the loading control. **D** IC_50_ values of cisplatin, carboplatin, and oxaliplatin were estimated in MDA-MB-231 stable cells (Con; miR-con and 320c; miR-320c). The table of each measurement described the IC_50_ values. Data are presented as mean ± SD. **E**, **F** MDA-MB-231 stable cells (Con; miR-con and 320c; miR-320c) were plated in six wells (1000 cells/well), following which 3′DW (triple-distilled water), cisplatin, carboplatin, and oxaliplatin were used for treatment. After 7–14 days of incubation, crystal violet staining of colonies was performed. Representative images are shown. The graph showed the relative clonogenic survival rate in each treated group. **G**–**I** miR-320c expression levels in Hs578T stable cell lines (Con; miR-con and 320c; miR-320c), as determined using TaqMan qRT-PCR. Con; control miRNA overexpressing Hs578T stable cell line, 320c; miR-320c overexpressing Hs578T stable cell line. CHK1 mRNA and protein levels were detected by qRT-PCR and western blot analysis. RNU48 and 18s rRNA were used for endogenous control. β-actin is used as a loading control. **J**, **K** Hs578T stable cells (Con, 320c) were plated in six wells (1000 cells/well) and then treated with 3′DW (triple-distilled water) or oxaliplatin. After 7–14 days of incubation, crystal violet staining of colonies was performed. Representative images are shown. The graph shows the relative colony number in each treatment group. All data are representative of three independent experiments. *P < 0.05, **P < 0.0.01, ***P < 0.001.

As the importance of Chk1 in the DDR is well-documented, we next tested if miR-320c regulation could affect the responsiveness of platinum-based drugs, which induce DNA damage to cells. We investigated which drugs were most effective against miR-320c overexpression. miR-320c expression levels successfully increased, and both Chk1 mRNA and protein were inhibited (Fig. 3A–C). To determine platinum-based reactivity, we measured IC_50_ values for cisplatin, carboplatin, and oxaliplatin in miR-320c overexpressing cells compared with those in control cells. As shown in Fig. 3D, the IC_50_ of oxaliplatin was significantly reduced, while that for cisplatin and carboplatin was marginally altered following miR-320c overexpression. In MDA-MB-231 and Hs578T cells, we observed that the viability was significantly inhibited by treatment oxaliplatin (50 μM) (Supplementary Fig. 5A). Additionally, the colony-formation assay revealed that oxaliplatin treatment decreased miR-320c cells’ survival compared with that of control cells (Fig. 3E, F). Next, these results were confirmed in Hs578T cells by generating stable cell lines (miR-Con and miR-320c). In Hs578T cells, Chk1 expression successfully decreased when miR-320c was overexpressed (Fig. 3G–I). The clonogenic assay showed a significant reduction in colony-formation capability with miR-320c overexpression. Moreover, oxaliplatin-treated miR-320c overexpressed cells were shown the most effective oxaliplatin response in each group (Fig. 3J, K). These results suggested that increased expression of miR-320c regulates the Chk1, thereby increasing the oxaliplatin’s effectiveness in TNBC cell lines.

### Upregulation of miR-320c increased apoptosis by modulating Chk1

**Fig. 4:**
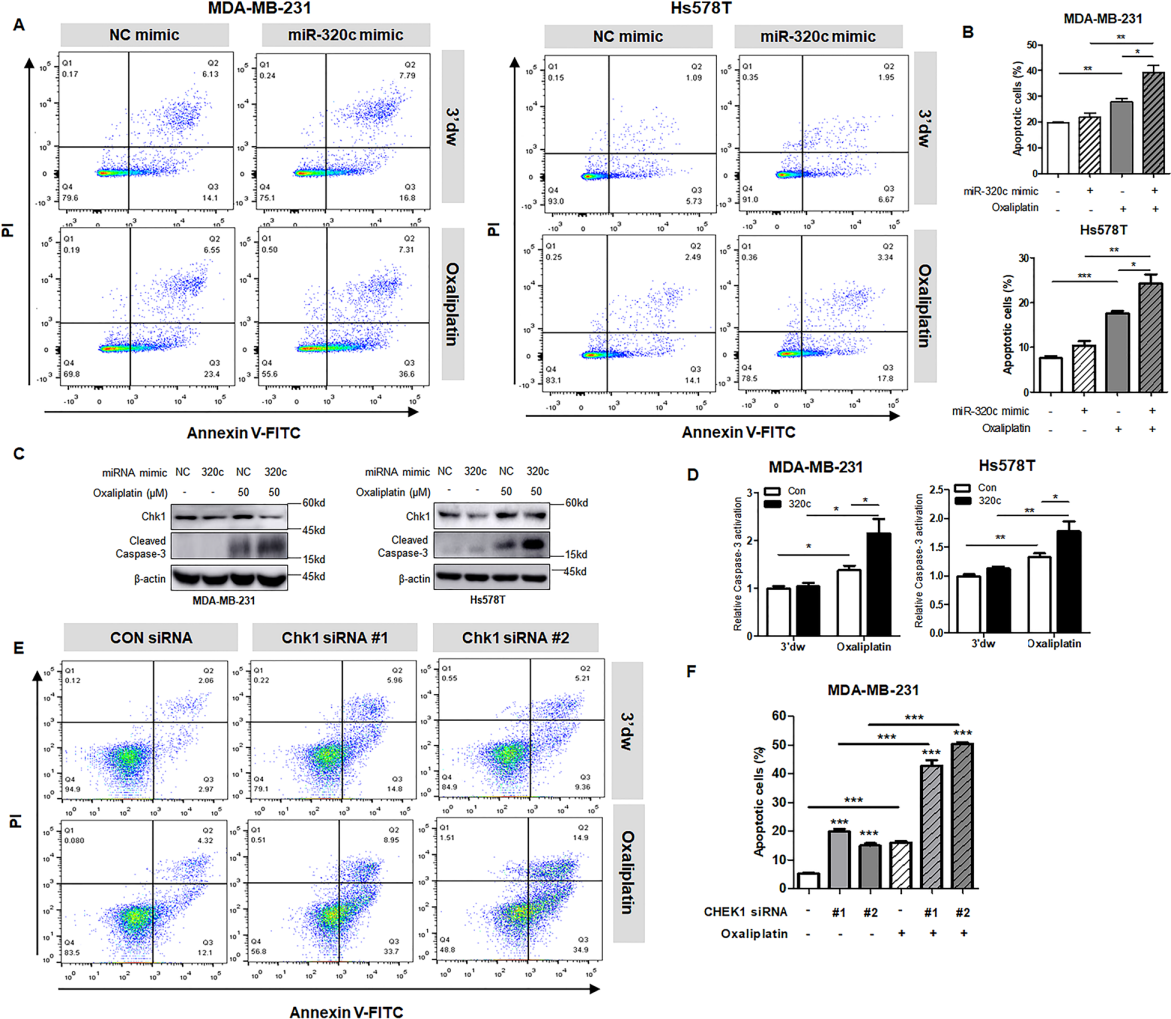
Upregulation of miR-320c increase apoptosis by modulating Chk1 expression. **A**, **B** MDA-MB-231 and Hs578T cells were transfected with negative control (NC) mimic or miR-320c mimic, and oxaliplatin treatment (50 μM) was followed for 48 h. Apoptotic cells were analyzed by flow cytometry. PI and FITC staining were used. The graph shows the mean ± SD of the apoptotic cell population in three independent experiments. **C** Chk1 and cleaved caspase-3 levels, as detected by treatment with the miR-negative control (NC) or miR-320c mimic (320c) and oxaliplatin in MDA-MB-231 and Hs578T cells. **D** Relative caspase-3 activation after incubation with oxaliplatin for 48 h in MDA-MB-231/miRNA-control (Con) or MDA-MB-231/miR-320c (320c) stable cells and Hs578T/miR-control (Con) or Hs578T/miR-320c (320c) stable cells. The data are representative of three independent experiments. **E**, **F** MDA-MB-231 cells with control siRNA or two types of Chk1-siRNA transfection were treated with oxaliplatin for 72 h. The two siRNAs targeted different sites. Apoptotic cells were analyzed by flow cytometry. PI and FITC staining were performed. The graph shows the mean±SD of three independent experiments. *P < 0.05, **P < 0.0.01, ***P < 0.001.

Next, we wanted to determine whether miR-320c modulates oxaliplatin responsiveness by regulating the DDR mediated by Chk1. In previous studies, it has been found that Chk1 phosphorylates various effectors involved in checkpoints, DNA repair, and apoptosis^16^. We investigated whether transfection of miR-320c with oxaliplatin could induce apoptosis by controlling the expression of Chk1. As a result, there was significantly increased apoptosis in TNBC cells treated with oxaliplatin and the miR-320c mimic compared to the other group (Fig. 4A, B). The other way to investigate cell apoptosis in each group was to measure cleaved caspase-3 after overexpressing miR-320c and incubation with oxaliplatin in MDA-MB-231 and Hs578T cells. Levels of cleaved caspase-3 were also highly increased by treating of oxaliplatin and the miR-320c mimic compared to the miR-control-treated group. Likewise, caspase-3 activation was highly elevated in stably miR-320c overexpressed cells treated with oxaliplatin in TNBC cells. These results suggested that Chk1 expression was reduced, and oxaliplatin-induced apoptosis was induced by increasing miR-320c expression. Furthermore, we confirmed that the effects of miR-320c on apoptosis were the result of reduced Chk1 expression. Similarly, when Chk1 levels were reduced, the apoptosis ratio was increased. Besides, the Chk1-siRNA transfected- and oxaliplatin-treated group’s apoptosis ratio was increased twofold to tenfold compared to the other groups (Fig. 4E, F). Therefore, these findings established that miR-320c with oxaliplatin-induced apoptosis suppressed the expression of Chk1 in TNBC cells.

### Upregulated miR-320c induces DNA damage accumulation by inhibiting Chk1 expression

**Fig. 5:**
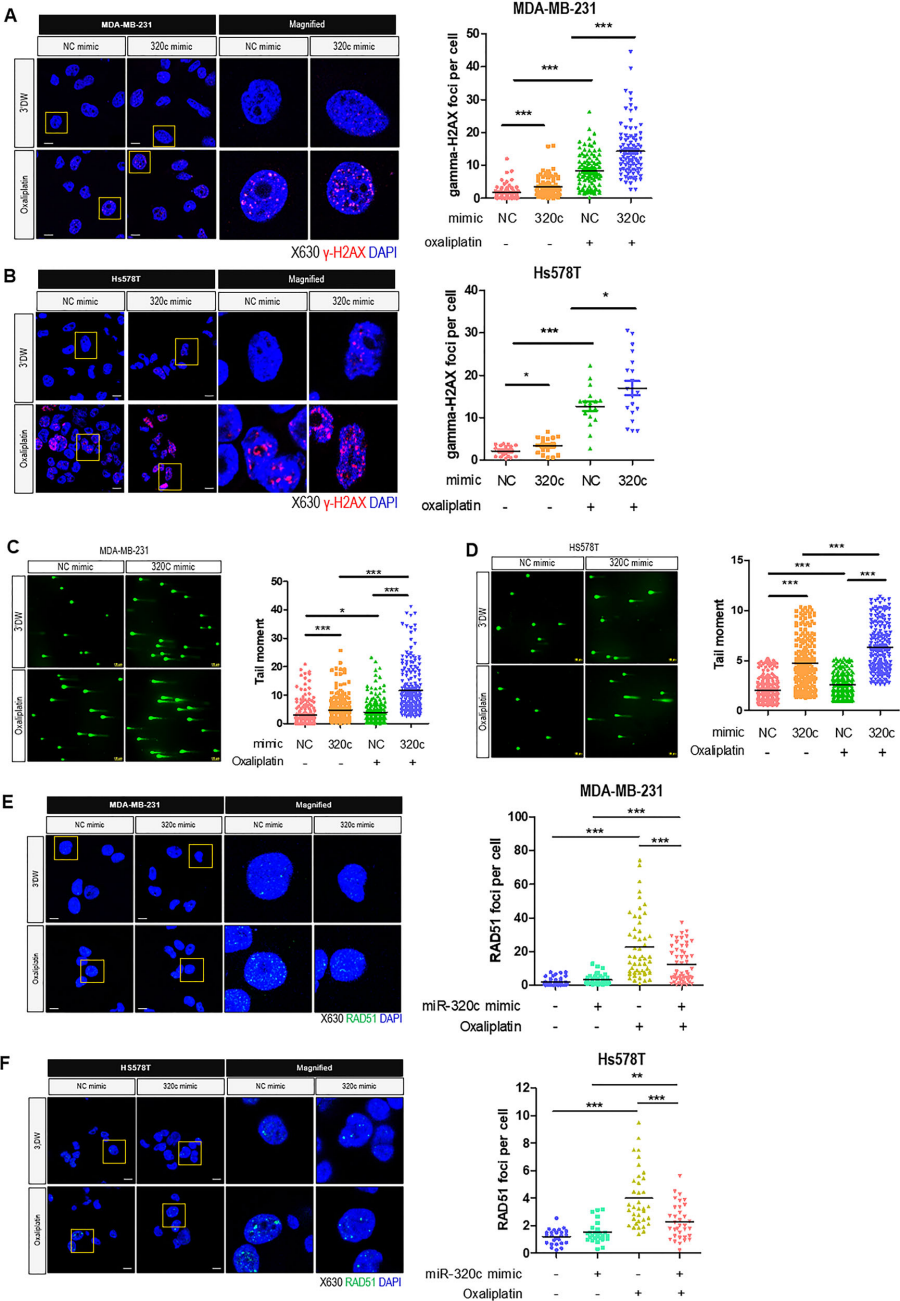
Increase of miR-320c induce DNA damage accumulation by regulating Chk1. **A**, **B** Representative images of MDA-MB-231 and Hs578T cells with negative control (NC) mimic or miR-320c mimic transfection and oxaliplatin treatment (left). Each sample was stained for nuclei (DAPI; blue) and non-repaired DNA damage (gamma-H2AX; red). Scale bar = 10 μm. The percentage of damaged cells is shown (right). Data were representatively shown as mean ± SD of at least 100 cells in every four independent experiments. **C**, **D** Alkaline comet assay performed on MDA-MB-231 cells and Hs578T treated with 250 μM oxaliplatin or 3′DW (triple-distilled water) for 1 h followed by 48-h repair interval (left). The representative image is shown. Scale bar = 100 μm. Tail moments were measured using a microscope and expressed as the mean ± SD of at least 100 cells in each treatment group. The graph showed the tail moments in each group (right). **E**, **F** Representative images of RAD51 foci formation in MDA-MB-231 cells and Hs578T cells. Cells were transfected with miR-NC mimic or miR-320c mimic and were treated with 3′DW (triple-distilled water) or oxaliplatin (left). Each sample was stained for nuclei (DAPI; blue) and RAD51 (green). Scale bar = 10 μm. The foci numbers per each cell are shown (right). Data represent mean ± SD of at least 100 cells in every four independent experiments. *P < 0.05, **P < 0.0.01, ***P < 0.001.

It was previously demonstrated that DNA damage might induce homologous recombination repair (HRR) by RAD51 phosphorylation through Chk1^16^. Previous studies showed that inhibition of Chk1 causes DNA damage and H2AX phosphorylation^34,35^. We attempted to determine the role of miR-320c in the oxaliplatin-induced DNA damage repair by regulating Chk1 expression. First, to investigate the requisite dose to induce DNA damage, TNBC cells were treated with various oxaliplatin concentrations for 1 h, followed by 48 h for repair. Based on the viability results, we determined the appropriate dose to induce DNA damage in both MDA-MB-231 and Hs578T cells (Supplementary Fig. 5B). We stained with γ-H2AX (phosphorylated H2AX), a DNA double-strand break marker, and checked γ-H2AX foci to detect unrepaired DNA damage. As we expected, more damage was observed in miR-320c-overexpressing-TNBC cells with the oxaliplatin treatment group than other groups (Fig. 5A, B). In addition, we performed the alkaline comet assay to detect DNA breaks, which are induced by DNA damage in MDA-MB-231 and Hs578T TNBC cells. The cells were transfected with miR-320c or NC (negative control). As a result, when oxaliplatin was administered, miR-320c-transfected cells exhibited increased-DNA-damage, compared with the NC-transfected cells (Fig. 5C, D).

Furthermore, we stained and detected RAD51 in cells because RAD51 is recruited to DNA that is damaged^34^. When oxaliplatin was administered, RAD51 foci formation was increased. However, miR-320c transfection decreased RAD51 foci in oxaliplatin-treated TNBC cells (Fig. 5E, F). In addition, the decrease in RAD51 foci formation was detected in Chk1-siRNA transfected TNBC cells (Supplementary Fig. 6). These results indicated that the upregulation of miR-320c inhibited the HRR of oxaliplatin-induced DNA damage in TNBC cells by inhibiting Chk1.

### miR-320c upregulation increases drug response by modulating Chk1, in vivo

Based on the in vitro studies, to suggest the effect on apoptosis and DNA damage repair of miR-320c upregulation with oxaliplatin in TNBC, we generated an in vivo xenograft model. First, MDA-MB-231/A cells were subcutaneously injected into nude mice to generate a xenograft mouse model. When the tumors were generated, they were subjected to oxaliplatin (intraperitoneal) and miR-320c mimic (intra-tumorigenic). After 3 weeks, the mice were sacrificed, and the xenografts tumor were obtained.

**Fig. 6:**
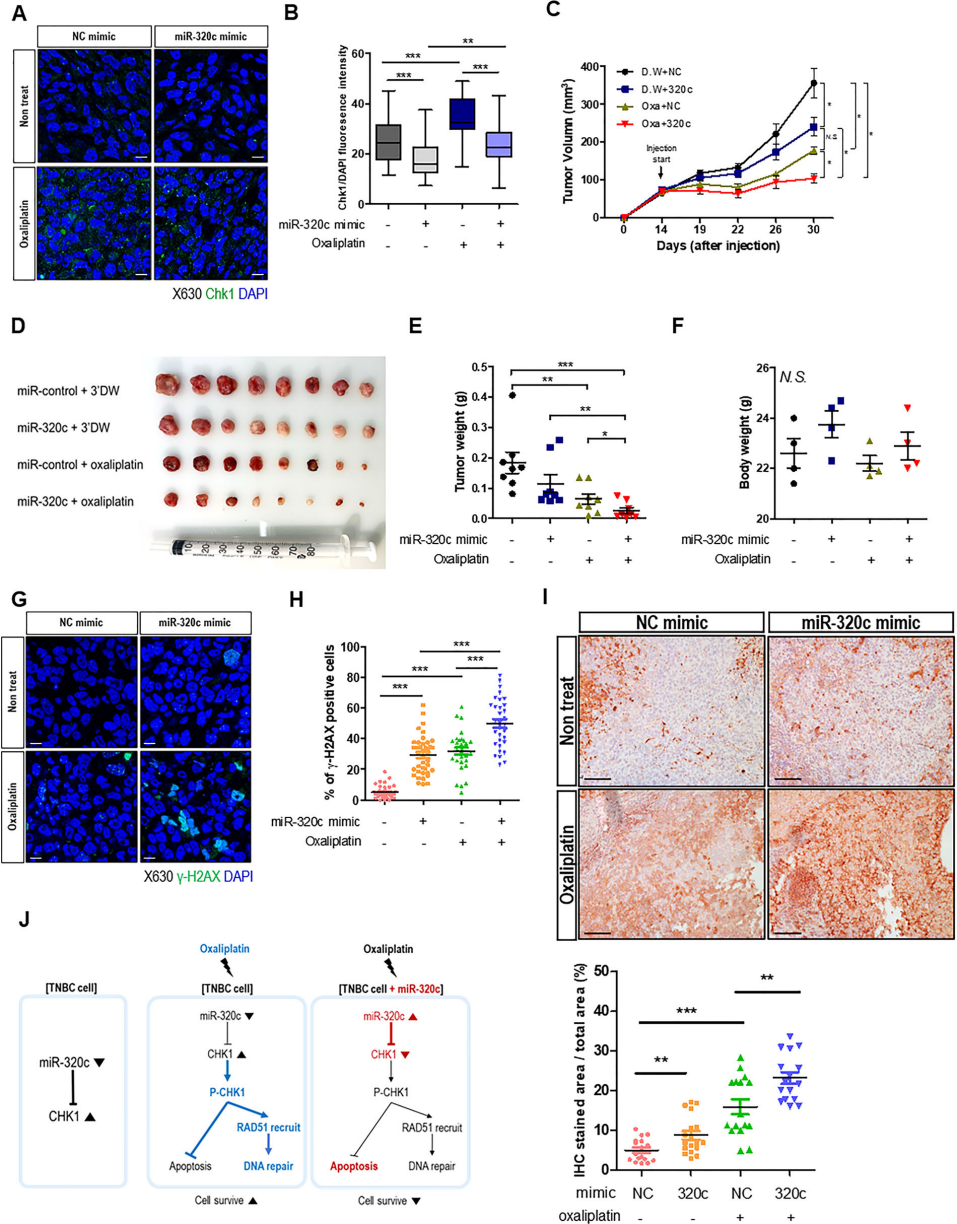
miR-320c mimic downregulates the drug response, in vivo. **A** Immunohistochemical staining was used to detect Chk1 expression in xenograft tumors. Each sample was stained for nuclei (DAPI; blue) and Chk1 (green). The representative images are shown. Scale bar = 10 μm. **B** ICC intensity scores of Chk1 staining from NC mimic- and 3′DW-treated, NC mimic- and oxaliplatin-treated, miR-320c mimic- and 3′DW (triple-distilled water)-treated, and miR-320c mimic- and oxaliplatin-treated xenograft mouse tumors. Data are presented as mean ± SEM. **C** MDA-MB-231/A cells were injected subcutaneously into nude mice, then NC (negative control) mimic and miR-320c mimic are injected in the tumor directly twice per week. Tumor volumes were measured every 3–5 days. The tumor volume of each group of mice was followed up for 30 days. Data are presented as mean ± SEM. **D** The tumor size of each group was shown. **E** After mice sacrificed, tumor weight was measured. **F** Before the mice sacrificed, the bodyweight of each group was measured. N.S. means statically non-significant. **G**, **H** Immunohistochemical staining was used to detect γ-H2AX, and the histogram presents the percentage of γ-H2AX-positive cells. Each sample was stained for nuclei (DAPI; blue) and γ-H2AX (green). The representative images are shown. γ-H2AX; gamma-H2A histone member X. Scale bar = 10 μm. **I** Immunohistochemical staining (cleaved caspase-3) was used for assessing apoptosis in each sample. Representative images are shown (upper). The stained area of cleaved caspase-3 per total area (%) is described in the bar graph (bottom). γ-H2AX, gamma-H2A histone member X. Scale bar = 100 μm. **J** Chk1 is regulated by miR-320c in triple-negative breast cancer (TNBC). Chk1 activation induces DDR in oxaliplatin-treated TNBC cells (upper). The effect of miR-320c on oxaliplatin-induced Chk1 signaling in TNBC cells is described. *P < 0.05, **P < 0.0.01, ***P < 0.001.

First, to confirm that the effect of miR-320c on tumor progression suppression is due to Chk1 downregulation, we evaluated Chk1 levels using immunofluorescence (IF). The Chk1 levels were significantly downregulated by miR-320c mimic injection (Fig. 6A, B). When miR-320c downregulated the Chk1, the tumor mean volume was lower than that of NC-treated groups. Both miR-320c and oxaliplatin-treated groups showed significantly lower tumor volume than other groups (Fig. 6C, D). In addition, these patterns were observed in tumor weight (Fig. 6E), whereas bodyweight comparison between each group showed no significant difference (Fig. 6F). Furthermore, γ-H2AX and cleaved caspase-3 expression were significantly increased in both miR-320c- and oxaliplatin-treated tumor groups (Fig. 6G–I). To sum up these results, the upregulation of miR-320c by mimic injection enhanced oxaliplatin treatment.

We identified that miR-320c plays a role in the novel regulatory mechanism of Chk1 in TNBC (Fig. 6J). TNBC cells showed lower miR-320c expression and higher Chk1 levels. When TNBC cells were treated with oxaliplatin, Chk1 was successfully activated by induced DNA damage. Activated Chk1 suppressed DNA damage-induced apoptotic response and RAD51 recruitment to the DNA damage site for DNA repair. However, when miR-320c was increased, enough Chk1 could not be synthesized. As a result, increased apoptosis and decreased RAD51 recruitment were observed in miR-320c-upregulated TNBC cells compared to miR-320c-downregulated TNBC cells, leading to attenuation of DNA repair and accumulation of DNA damage. Collectively, miR-320c upregulation made TNBC cells more sensitive to oxaliplatin.

## Discussion

It has been reported that Chk1 is overexpressed in various cancer cells and Chk1 functions are critical for cancer cell survival^17^. For the first time, we showed that enhanced Chk1 expression in TNBC is due to the downregulation of miR-320c. Further biochemical analysis demonstrated that, indeed, Chk1 expression is regulated by miR-320c. Unlike other breast cancer subtypes, treatment of TNBC patients is limited to chemotherapy, radiotherapy, and surgical treatment, and the prognosis is the poorest one. Additionally, the heterogeneity of TNBC makes it challenging to diagnose and determine the appropriate treatment. Thus, it is essential to identify TNBC-specific molecular targets that are used as prognostic markers and therapeutic targets to increase the responsiveness toward drugs^36^. Here, we explored the potential role of miR-320c as a regulator of Chk1 and therapeutic target in a TNBC.

We investigated the abnormally reduced miR-320c in TNBC. Several reports have identified dysregulated miRNAs associated with disease models and have elucidated the regulatory mechanisms of altered expression. Transcriptional regulation is one molecular mechanism regulating miRNAs, including methylation of a promoter or dysregulated transcription factor such as PTEN and TP53, thus modulating miRNA expression^37^. Reportedly, the miR-320 family, including miR-320c, is regulated by PTEN^8^. Notably, PTEN loss is associated with the highly aggressive triple-negative phenotype in breast cancer^38^. Thus, it could be postulated that a dysregulated transcription factor, such as PTEN, could regulate the expression of miR-320c in TNBC. Nevertheless, the precise mechanism regulating the reduced miR-320c expression in TNBC needs to be elucidated.

This study showed that Chk1 is upregulated in TNBC, and in vitro experiments suggest that miR-320c plays an evident suppressive role in Chk1 expression in TNBC cells. miRNAs have advantages over siRNA in diseases that require the adjustment of various pathways for effective treatment, such as cancers. For these reasons, in various studies, it has been shown that synthesized miRNAs or miRNA mimics have demonstrated therapeutic effects^39,40^. Especially, miRNA replacement therapy has been proposed in several investigations. In cancer chemotherapy, miRNA mimics have several advantages. First, the size of miRNAs is substantially small, and hence miRNA mimics could effectively enter the cytoplasm to participate in delivery to a target tissue. Moreover, miRNA mimics were designed to allow therapeutics to possess the same sequence as naturally occurring miRNAs, and are expected to regulate their counterparts to interact with target genes effectively, rarely presenting off-target effect. Additionally, when target genes of miRNA are suppressed by endogenously deregulated miRNA, miRNA mimics for treatment selectively influence cancer cells, with fewer effects on normal cells^41^. Hence, miRNA replacement therapy can specifically influence cancer cells. Previous studies reported that targeting Chk1 by using a Chk1 inhibitor in TNBC is therapeutically beneficial^42^. However, pharmacological inhibition of Chk1 activity reportedly induces adverse effects, including cardiac toxitiy^43^. Inhibition of protein activity could control and affect both types of cells, normal as well as cancer cells. Numerous studies also suggested that combination with miRNAs and anticancer drug treatments for therapeutics could show a synergetic effect on tumor cells selectively^44,45^. Therefore, we propose miR-320c as a potential treatment strategy in combination with anticancer drugs, such as oxaliplatin.

Platinum-based drugs are DNA-damaging agents and emerging therapies in early-stage TNBC and are used to treat a wide range of solid tumors, like colorectal cancer^46^. When three kinds of platinum-based drugs were used to induce DNA damage, miR-320c overexpression significantly promoted increased oxaliplatin responsiveness. Oxaliplatin induces intrastrand links between two adjacent guanine residues or between a guanine and an adenine residue. These links interrupt DNA replication and transcription, resulting in DNA damage. Owing to the DACH carrier ligand, oxaliplatin reacts efficiently in cisplatin- or carboplatin-resistant cells. Especially, oxaliplatin is used to treat colorectal cancer, which intrinsically presents cisplatin- and carboplatin resistance^47^. Oxaliplatin differs from cisplatin in terms of DNA binding, adduct formation, and apoptosis^15^. However, the distinct molecular mechanisms underlying the effects of cisplatin, carboplatin, and oxaliplatin warrant further investigation. A few studies have investigated the effects of oxaliplatin in advanced-stage breast cancer^48^. As metastatic TNBC (mTNBC) is known to present a poor prognosis, the standard of care includes taxanes; a cisplatin/carboplatin containing regimen as first-line therapy has demonstrated progression-free survival (PFS) of 3 months with single-agent treatment in mTNBC patients^49^. Hence, oxaliplatin could be considered in patients pretreated with cisplatin/carboplatin.

Therefore, we conducted experiments to suggest inhibition of Chk1 by upregulating miR-320c could enhance the responsiveness of oxaliplatin in TNBC, in vitro, and in vivo. Previous studies comparing GI_50_ (growth inhibition of 50%) to identify oxaliplatin sensitivities in the NCI-60 cell lines revealed that the TNBC cell line (MDA-MB-231 and HS578T) showed higher GI_50_ than non-TNBC cell line (MCF7 and T47D)^50^. Furthermore, while low levels of miR-320c were significantly associated with poor survival in the TNBC subtype, high levels of miR-320c were significant in the Luminal A and Her2 subtypes. miR-320c has previously been shown not to be deregulated in BRCA1/2-associated breast carcinomas^51^. Given the results of this study and the importance of Chk1 in the DDR, we suggested that miR-320c was responsible for the difference in Chk1 levels seen in TNBC cells, and that this caused the differences in response to DNA-damaging agents. Moreover, these data indicated that decreased miR-320c expression and upregulated Chk1 expression, like the BRCA1 mutation, could serve as useful prognosis markers for decisions regarding treatment with oxaliplatin. Our findings suggested that miR-320c could be a potential prognostic marker for developing a treatment strategy in mTNBC or TNBC patients.

As summarized in Fig. 6J, DNA damage induced by oxaliplatin activates Chk1, and activated Chk1 is involved in DDR pathways to repair DNA damage. However, when combined with the increase in miR-320c and oxaliplatin treatment in TNBC cell lines, Chk1 could not be sufficiently activated because of the miR-320c effect, and unrepaired DNA damages accumulated, resulting in suppressed tumor progression. Collectively, we suggest that miR-320c can be a potential treatment method with oxaliplatin and a potential prognostic marker to decide for an oxaliplatin treatment for TNBC patients.

## Materials and methods

### Cell culture

HEK293T, SK-BR-3, MCF7, MDA-MB-468, Hs578T, and MDA-MB-157 cells were cultured in Dulbecco’s modified Eagle’s medium (DMEM, WelGENE, South Korea), and MDA-MB-231, HCC-1937, ZR-75-1 cells were cultured in Roswell Park Memorial Institute (RPMI) 1640 Medium (RPMI, WelGENE). The culture medium was supplemented with 10% fetal bovine serum (FBS, Gibco, Carlsbad, CA, USA) and 1% penicillin/streptomycin (WelGENE). MCF-10A cells were cultured in a growth medium consisting of DMEM:F12 (1:1) in 5% horse serum (Invitrogen, Carlsbad, CA, USA), and supplemented with 20 ng/mL EGF, 0.5 mg/mL hydrocortisone, 100 ng/mL cholera toxin, and 10 µg/mL insulin (Sigma-Aldrich, St. Louis, MO, USA). Moreover, all cell lines were authenticated by short tandem repeat (STR) marker profiling (Cosmo Genetech Co., Ltd., Korea) and verified as mycoplasma-free using the e-Myco™ VALiD Mycoplasma PCR Detection Kit (iNtRon Biotechnology, Korea).

### Quantitative RNA isolation and quantitative RT-PCR (qRT-PCR)

Synthesized cDNA was used for qPCR using SYBR Green qPCR Master Mix (PCR Biosystem, London, UK). Each target gene was detected by using a specific primer. PCR primers are described in Supplementary Table 2.

### Dual-luciferase reporter assay

Human Chk1 3′-UTR was amplified by PCR from MDA-MB-231 cDNA. The sequence of primers used to amplify the wild-type (WT) and mutant-type (MT) gene is shown in Supplementary Table 3. These luciferase constructs were co-transfected in HEK293T cells with miR-320c mimic or miR-NC mimic, using Lipofectamine 2000 reagents (Invitrogen). Luciferase activities were measured using the Dual-Luciferase Reporter Assay System (Promega), according to the manufacturer’s manual.

### Analysis of human breast tumor microarray data

Analysis of miRNA microarray data in TNBC and adjacent normal tissues was previously described^19^. Briefly, breast cancer samples were surgically resected from patients after approval by the Institutional Review Board of Seoul National University Hospital (IRB number: 1704-019-843). The tumor specimen and normal tissue were acquired from the same patient’s tumor core. For normal tissue, the specimen was obtained at least 5 cm from the cancer lesion. Using H&E staining and section examination, we determined the absence of cancer cells in normal tissues. Tissues were stored in nitrogen tanks at a tissue bank (Laboratory of Breast Cancer Biology, Seoul National University College of Medicine, Seoul, Korea). Informed consent was obtained from all patients before collection of specimens. The total RNA purified from each specimen was used for microRNA microarray. The value of the probe signal was transformed into the logarithm and normalized using a quantile method.

### Immunostaining (ICC/IF/IHC) microscopy

For immunocytochemistry (ICC), cells incubated on coverslips were fixed by 10% neutral buffered formalin (Biosesang, Seongnam, Korea). After then, the cell was permeabilized and incubated with anti-phospho-histone H2A.X (Ser139) antibody (Merck Millipore, Darmstadt, Germany) or anti-Rad51 antibody (Abcam, Cambridge, UK) at 4 °C overnight. For IF, paraffin sections of xenograft tumors were used. The slides were incubated with the Chk1 antibody (Abcam) or anti-phospho-Histone H2A.X (Ser139) antibody (Merck Millipore) at 4 °C overnight. For immunostaining microscopy, images were visualized by confocal laser scanning microscope (LSM 700, Carl Zeiss, Oberkochen, Germany), and each image was captured with identical exposure settings. For immunohistochemistry, staining with cleaved caspase-3 antibody (Cell Signaling Technology, Beverly, MA, USA) was performed using Vectastatin ABC kit (Vector Laboratories, Burlingame, CA, USA) according to the manufacturer’s instruction. Staining was visualized by NovaRED substrate (Vector Laboratories), and counterstaining was performed with hematoxylin.

### Western blotting

Whole-cell lysates were prepared in RIPA buffer (Thermo Scientific, Rockford, IL, USA). Primary Antibodies used in this study were Chk1 (Santa Cruz Biotechnology, Dallas, TX, USA) and β-actin (Bethyl laboratories, Montgomery, TX, USA). The raw data are shown in Supplementary Fig. 7.

### Alkaline comet assay

The DNA damage was detected using the alkaline comet assay kit according to the manufactures’ protocol (4250-050-K, Trevigen, Gaithersburg, MD, USA). Slides were wash and stain with SYBR for 30 min. Images were obtained by Confocal Microscope (Nikon, Japan). Tail moments were scored by an image analyzer system (Comet Assay IV, Version 4.3.2, Perceptive Instruments Ltd., Suffolk, UK).

### Cell viability assay

For determination of the IC_50_ value, cells (MDA-MB-231/miR-control and MDA-MB-231/miR-320c) were seeded and incubated with various concentrations of cisplatin (sigma), carboplatin (sigma), and oxaliplatin (Sigma) for 48 h. Then, the cell viability was detected using the Cell Counting Kit-8 (Dojindo Technologies, MD, USA) as following the manufacturer’s protocol. The IC_50_ values were calculated by GraphPad Prism 5.0. Hs578T and MDA-MB-231 cells were transfected with miRNA for 24 h and then treated with oxaliplatin for 48 h. After that, the CCK-8 labeling mixture was added to the cells of each group and incubated for 1 h. The absorbance at 450 nm was measured using the spectrophotometer (Synergy HTX, BioTek Instruments).

### Apoptosis analysis (FACS)

The prepared cells were trypsinized and washed in cold PBS. Then the cells were stained with FITC and PI by FITC Annexin V Apoptosis Detection Kit I (BD Pharmingen™, San Diego, CA, USA). After then, cells were analyzed by Flow Cytometer (FACS Canto II, BD BioSciences, San Jose, CA, USA). A total of 10,000 cells were counted for each sample.

### Caspase-3 activation assay

Cells which show stable overexpression of miR-320c and miR-control cell were used. The oxaliplatin (Sigma) was treated to each plate for 48 h. After incubation, cells were harvested, and the subsequent procedures were carried out by ApoAlert Caspase Colorimetric Kit (Clontech Laboratories, Mountain View, CA, USA).

### Xenograft mice and intratumoral miRNA transfection

All animal experiments were approved by the Institutional Review Board of the Yonsei University College of Medicine and were performed in specific pathogen-free facilities according to the university’s guidelines for the Care and Use of Laboratory Animals (2018–0155). Thus, 8-week-old female Balb/c-nude mice (Orient, Seongnam, Korea) were inoculated subcutaneously with 2.0 × 10^6^ MDA-MB-231/A cells into each flank under 100 µL of saline/zoletil/rompun (7:1:1) anesthesia. When tumors reached an average volume of 100–150 mm^3^, after 2 weeks, the mice were then treated with intratumoral injection of synthetic miRNA and intraperitoneal (IP) injection of oxaliplatin (Tocris Bioscience, Bristol, UK) or 3′DW (3rd distilled water). Then, 3′DW or oxaliplatin was applied with an intraperitoneal injection (2 mg/kg) in 3–5-day intervals.

### Statistical analyses

Data are presented as means ± SEM or SD. A Student’s *t* test was used for statistical analysis using GraphPad Prism 5 software (GraphPad, USA). A *P* value of <0.05 compared with control was considered statistically significant. Asterisks indicate statistical significance as determined by the independent samples *t* test. *P < 0.05; **P < 0.01; ***P < 0.001.

## Supplementary information

Supplementary figures and table

Supplementary Materials and methods
