# Supplementary material for: Inhibition of Chk1 by miR-320c increases oxaliplatin responsiveness in triple-negative breast cancer
Source: Oncogenesis. 2020 Oct 11;9(10):91. doi: 10.1038/s41389-020-00275-x (PMC7548284; doi:10.1038/s41389-020-00275-x)
Supplement: Supplementary file 1 — Supplementary figures and table [file 41389_2020_275_MOESM1_ESM.docx]

| ProbeID | N1 | N2 | N3 | C1 | C2 | C3 | TNBC C/TNBC N.  fold change | TNBC C/TNBC N  .raw.pval |
| --- | --- | --- | --- | --- | --- | --- | --- | --- |
| hsa-let-7d-5p | 8.772935 | 9.162556 | 8.833943 | 10.362847 | 10.023183 | 9.47359 | 2.042112 | 0.069665878 |
| hsa-let-7i-5p | 8.096935 | 9.59294 | 8.701416 | 9.345438 | 10.140817 | 10.483344 | 2.285912 | 0.079228122 |
| hsa-miR-100-5p | 9.125694 | 10.546623 | 9.767683 | 8.937113 | 7.445617 | 8.962899 | -2.575389 | 0.263421658 |
| hsa-miR-101-3p | 3.188537 | 7.195116 | 5.450471 | 5.8073 | 8.313323 | 7.74321 | 4.027551 | 0.047747939 |
| hsa-miR-103a-3p | 7.828748 | 8.903215 | 8.023337 | 10.235372 | 10.593723 | 9.849133 | 3.929402 | 0.012153095 |
| hsa-miR-106b-5p | 3.844535 | 6.450958 | 5.251636 | 9.125694 | 9.732171 | 8.143906 | 14.105772 | 0.035573119 |
| hsa-miR-107 | 7.547813 | 8.401502 | 7.595952 | 10.140817 | 10.280033 | 9.308811 | 4.1741 | 0.016729895 |
| hsa-miR-10a-5p | 8.213151 | 8.374865 | 8.527994 | 3.100095 | 5.589661 | 7.796446 | -7.344187 | 0.150946593 |
| hsa-miR-10b-5p | 9.47359 | 9.767683 | 9.633341 | 5.470725 | 7.467433 | 8.462317 | -5.623155 | 0.094001363 |
| hsa-miR-1181 | 4.511562 | 3.844535 | 3.813837 | 4.549473 | 6.258087 | 4.843926 | 2.235376 | 0.234097376 |
| hsa-miR-1207-5p | 10.054638 | 9.443075 | 10.235372 | 8.527994 | 8.462317 | 8.801254 | -2.486024 | 0.016084586 |
| hsa-miR-1224-5p | 6.551124 | 5.741398 | 6.20364 | 4.963722 | 4.985888 | 4.775532 | -2.389994 | 0.038755417 |
| hsa-miR-1227-5p | 9.890922 | 7.957741 | 10.095662 | 7.259853 | 4.316308 | 6.285686 | -10.273291 | 0.011784952 |
| hsa-miR-1246 | 7.979241 | 6.890674 | 7.828748 | 9.633341 | 7.617967 | 9.23086 | 2.396898 | 0.0449093 |
| hsa-miR-125a-3p | 6.954691 | 6.043943 | 7.796446 | 5.636289 | 5.297637 | 5.855749 | -2.522991 | 0.060715548 |
| hsa-miR-125b-5p | 12.301248 | 13.579186 | 13.128511 | 11.562278 | 9.398339 | 11.284945 | -4.771561 | 0.156373723 |
| hsa-miR-1260a | 12.069148 | 11.562278 | 10.483344 | 13.128511 | 13.128511 | 13.128511 | 3.379761 | 1 |
| hsa-miR-1260b | 11.956518 | 10.801181 | 10.362847 | 13.579186 | 11.672532 | 12.301248 | 2.784607 | 0.042946366 |
| hsa-miR-1275 | 7.259853 | 6.658416 | 7.766717 | 6.703109 | 5.470725 | 6.095311 | -2.201694 | 0.071786401 |
| hsa-miR-128 | 2.280774 | 4.46582 | 4.13036 | 6.634618 | 6.720936 | 4.940824 | 5.552515 | 0.138063048 |
| hsa-miR-1290 | 5.415786 | 4.181575 | 4.733905 | 8.632122 | 5.783729 | 7.617967 | 5.927884 | 0.034829983 |
| hsa-miR-134 | 6.75305 | 5.835648 | 6.658416 | 5.251636 | 4.898711 | 5.201302 | -2.45971 | 0.018927914 |
| hsa-miR-135a-3p | 3.295114 | 3.902367 | 2.792112 | 4.44218 | 4.44218 | 4.668421 | 2.277939 | 0.091516391 |
| hsa-miR-140-3p | 6.929097 | 7.907458 | 7.302799 | 10.715316 | 7.490056 | 7.034578 | 2.04703 | 0.531206497 |
| hsa-miR-140-5p | 4.276879 | 5.936221 | 5.106172 | 8.903215 | 6.432406 | 6.68199 | 4.700387 | 0.212761469 |
| hsa-miR-145-5p | 11.562278 | 11.672532 | 11.476493 | 6.616997 | 8.557467 | 10.140817 | -8.766536 | 0.095156627 |
| hsa-miR-146a-5p | 4.109426 | 6.470331 | 5.157027 | 5.086088 | 8.427419 | 5.888627 | 2.332349 | 0.082454611 |
| hsa-miR-148a-3p | 6.06746 | 8.056692 | 7.259853 | 11.476493 | 8.213151 | 9.59294 | 6.202543 | 0.226136016 |
| hsa-miR-148b-3p | 2.61334 | 4.775532 | 3.718493 | 6.380205 | 7.394503 | 6.002636 | 7.41267 | 0.023294045 |
| hsa-miR-149-5p | 4.756836 | 4.489171 | 4.208109 | 5.157027 | 8.26591 | 4.549473 | 2.840408 | 0.315891246 |
| hsa-miR-150-5p | 9.802901 | 9.308811 | 8.903215 | 2.61334 | 11.210763 | 6.380205 | -6.07776 | 0.42578039 |
| hsa-miR-151a-3p | 4.571186 | 5.636289 | 4.92255 | 8.234415 | 6.492344 | 5.8073 | 3.485449 | 0.192659279 |
| hsa-miR-151a-5p | 7.213627 | 7.979241 | 7.445617 | 10.801181 | 8.291452 | 7.649618 | 2.580985 | 0.343114997 |
| hsa-miR-151b | 5.936221 | 7.054346 | 6.470331 | 9.23086 | 7.338143 | 6.774293 | 2.452295 | 0.325006884 |
| hsa-miR-1587 | 8.164983 | 6.75305 | 8.772935 | 6.131235 | 5.251636 | 5.835648 | -4.461357 | 0.035723645 |
| hsa-miR-15a-5p | 5.681177 | 6.524909 | 5.888627 | 7.369709 | 9.54856 | 8.937113 | 6.00802 | 0.028858274 |
| hsa-miR-15b-5p | 7.675637 | 8.427419 | 7.882515 | 9.512305 | 10.635118 | 8.714371 | 3.085294 | 0.05838809 |
| hsa-miR-16-5p | 9.23086 | 9.923926 | 9.308811 | 10.054638 | 11.956518 | 10.593723 | 2.603454 | 0.059360948 |
| hsa-miR-17-5p | 5.697637 | 7.467433 | 6.380205 | 8.557467 | 9.345438 | 7.445617 | 3.822234 | 0.064987949 |
| hsa-miR-181a-5p | 6.450958 | 7.796446 | 6.929097 | 9.54856 | 8.401502 | 9.512305 | 4.273116 | 0.110177008 |
| hsa-miR-181b-5p | 4.668421 | 5.201302 | 4.600237 | 7.338143 | 6.524909 | 6.634618 | 4.025692 | 0.035463548 |
| hsa-miR-186-5p | 3.3498 | 6.017615 | 4.650903 | 5.835648 | 7.103316 | 5.759976 | 2.948962 | 0.077872259 |
| hsa-miR-1914-3p | 5.106172 | 4.420248 | 4.668421 | 4.109426 | 2.61334 | 3.983577 | -2.238966 | 0.073670879 |
| hsa-miR-1915-3p | 10.199535 | 9.019009 | 9.59294 | 8.374865 | 8.164983 | 8.401502 | -2.445356 | 0.045354252 |
| hsa-miR-193a-3p | 4.818223 | 5.569962 | 4.316308 | 6.06746 | 8.604025 | 7.979241 | 6.271212 | 0.067081503 |
| hsa-miR-19b-3p | 6.182175 | 9.198185 | 7.175449 | 9.198185 | 10.546623 | 9.54856 | 4.743175 | 0.043704629 |
| hsa-miR-200b-3p | 5.741398 | 8.604025 | 7.394503 | 11.121826 | 9.849133 | 8.427419 | 5.86779 | 0.213010175 |
| hsa-miR-200c-3p | 6.231574 | 8.962899 | 7.213627 | 8.462317 | 10.317655 | 8.056692 | 2.782127 | 0.067763942 |
| hsa-miR-205-5p | 9.767683 | 12.506561 | 10.893592 | 5.297637 | 5.510864 | 8.903215 | -22.39917 | 0.089988161 |
| hsa-miR-20a-5p | 5.713913 | 8.143906 | 6.703109 | 8.746711 | 9.962742 | 8.023337 | 4.162029 | 0.056019756 |
| hsa-miR-20b-5p | 3.497679 | 6.352674 | 4.818223 | 7.034578 | 8.344674 | 6.703109 | 5.545285 | 0.043613735 |
| hsa-miR-21-5p | 8.833943 | 10.593723 | 9.54856 | 11.210763 | 13.579186 | 14.142637 | 9.97825 | 0.037497729 |
| hsa-miR-214-3p | 8.056692 | 8.023337 | 8.313323 | 4.92255 | 6.285686 | 9.198185 | -2.512245 | 0.376400587 |
| hsa-miR-2392 | 5.399292 | 4.511562 | 5.53103 | 3.497679 | 3.651221 | 3.154128 | -3.278309 | 0.062058139 |
| hsa-miR-24-3p | 8.714371 | 9.890922 | 8.962899 | 9.890922 | 10.199535 | 11.121826 | 2.32092 | 0.150961441 |
| hsa-miR-25-3p | 7.467433 | 8.119495 | 7.698118 | 8.801254 | 9.633341 | 7.94091 | 2.042242 | 0.121988416 |
| hsa-miR-27a-3p | 7.530381 | 9.398339 | 8.213151 | 9.264466 | 10.362847 | 11.210763 | 3.728862 | 0.085163982 |
| hsa-miR-27b-3p | 6.867295 | 8.291452 | 7.467433 | 10.095662 | 8.937113 | 10.362847 | 4.778244 | 0.108573413 |
| hsa-miR-28-5p | 3.983577 | 5.713913 | 4.940824 | 5.607302 | 6.634618 | 5.955637 | 2.275865 | 0.032802909 |
| hsa-miR-2861 | 11.284945 | 10.317655 | 11.956518 | 9.308811 | 7.595952 | 8.772935 | -6.178 | 0.017458944 |
| hsa-miR-29b-3p | 5.086088 | 7.74321 | 6.095311 | 8.056692 | 11.284945 | 9.694396 | 10.342226 | 0.003524228 |
| hsa-miR-29c-3p | 7.617967 | 10.362847 | 8.26591 | 8.19271 | 11.79004 | 10.199535 | 2.482603 | 0.080475057 |
| hsa-miR-30a-5p | 5.916385 | 8.67587 | 7.016099 | 9.47359 | 8.746711 | 8.19271 | 3.034697 | 0.259764924 |
| hsa-miR-30b-5p | 8.462317 | 10.140817 | 9.046876 | 11.79004 | 10.483344 | 9.090041 | 2.358394 | 0.359172929 |
| hsa-miR-30d-5p | 6.815317 | 8.234415 | 7.338143 | 10.280033 | 9.162556 | 7.567411 | 2.909374 | 0.257529898 |
| hsa-miR-30e-5p | 3.237845 | 6.285686 | 4.756836 | 8.67587 | 8.701416 | 7.907458 | 12.712059 | 0.056392382 |
| hsa-miR-320c | 7.717032 | 7.547813 | 7.649618 | 6.994715 | 6.043943 | 6.658416 | -2.10302 | 0.042780494 |
| hsa-miR-331-3p | 6.720936 | 6.634618 | 6.597104 | 7.766717 | 8.632122 | 7.882515 | 2.718665 | 0.037065734 |
| hsa-miR-34a-5p | 5.53103 | 7.828748 | 6.634618 | 7.567411 | 8.714371 | 10.023183 | 4.297576 | 0.100695729 |
| hsa-miR-3652 | 5.510864 | 5.415786 | 6.68199 | 4.756836 | 3.023483 | 4.13036 | -3.730374 | 0.080584791 |
| hsa-miR-3656 | 9.398339 | 8.313323 | 8.937113 | 6.890674 | 6.815317 | 6.720936 | -4.210376 | 0.020291207 |
| hsa-miR-3665 | 10.483344 | 9.345438 | 9.923926 | 8.143906 | 9.767683 | 8.492486 | -2.167769 | 0.303287286 |
| hsa-miR-3676-5p | 8.19271 | 7.675637 | 7.957741 | 10.199535 | 8.056692 | 9.046876 | 2.233034 | 0.132811341 |
| hsa-miR-3679-5p | 7.94091 | 6.774293 | 8.164983 | 6.470331 | 5.713913 | 6.043943 | -2.929525 | 0.037442126 |
| hsa-miR-371b-5p | 9.59294 | 7.850357 | 9.512305 | 7.175449 | 5.681177 | 6.352674 | -5.98811 | 0.01301863 |
| hsa-miR-374a-5p | 5.251636 | 6.703109 | 6.231574 | 6.658416 | 8.527994 | 8.344674 | 3.438045 | 0.012988221 |
| hsa-miR-375 | 3.419706 | 5.086088 | 2.892793 | 6.017615 | 5.201302 | 6.06746 | 3.89763 | 0.171659549 |
| hsa-miR-376a-3p | 2.972188 | 5.297637 | 4.882534 | 8.096935 | 3.154128 | 7.850357 | 3.953198 | 0.45464343 |
| hsa-miR-424-5p | 4.352069 | 6.095311 | 5.569962 | 8.870208 | 7.74321 | 8.527994 | 8.232649 | 0.067014392 |
| hsa-miR-425-5p | 5.35573 | 5.269257 | 5.062576 | 6.352674 | 8.119495 | 7.717032 | 4.491547 | 0.066339013 |
| hsa-miR-4257 | 5.759976 | 5.510864 | 5.551187 | 3.237845 | 5.336833 | 3.023483 | -3.343337 | 0.156366594 |
| hsa-miR-4271 | 6.492344 | 6.002636 | 6.450958 | 4.600237 | 6.002636 | 4.882534 | -2.224546 | 0.187051892 |
| hsa-miR-4286 | 8.632122 | 10.635118 | 8.056692 | 12.192239 | 12.927548 | 12.927548 | 11.91302 | 0.040734306 |
| hsa-miR-4291 | 3.54872 | 5.759976 | 5.00578 | 6.285686 | 6.994715 | 7.302799 | 4.256226 | 0.042642462 |
| hsa-miR-4298 | 5.201302 | 6.20364 | 6.06746 | 5.228466 | 2.972188 | 4.985888 | -2.691891 | 0.273879763 |
| hsa-miR-4299 | 10.317655 | 8.870208 | 9.962742 | 8.401502 | 7.766717 | 8.746711 | -2.660858 | 0.030923134 |
| hsa-miR-4306 | 4.940824 | 4.882534 | 5.415786 | 5.681177 | 7.547813 | 6.231574 | 2.652108 | 0.15490492 |
| hsa-miR-4324 | 6.977705 | 7.698118 | 7.103316 | 5.314363 | 3.100095 | 5.551187 | -6.081799 | 0.120650945 |
| hsa-miR-4327 | 5.314363 | 4.940824 | 6.131235 | 4.018337 | 4.155607 | 4.715047 | -2.243592 | 0.026442673 |
| hsa-miR-4433-3p | 6.131235 | 5.106172 | 5.713913 | 4.079654 | 4.181575 | 3.813837 | -3.085315 | 0.044100684 |
| hsa-miR-4449 | 4.861152 | 5.179711 | 4.400746 | 7.467433 | 5.314363 | 4.756836 | 2.04534 | 0.321143362 |
| hsa-miR-4463 | 5.589661 | 4.600237 | 5.228466 | 3.685683 | 4.650903 | 3.718493 | -2.17512 | 0.201098742 |
| hsa-miR-4466 | 9.54856 | 8.26591 | 9.125694 | 7.675637 | 8.234415 | 7.338143 | -2.346737 | 0.176763012 |
| hsa-miR-4497 | 8.313323 | 7.510092 | 7.675637 | 6.450958 | 7.034578 | 6.41167 | -2.298377 | 0.09603973 |
| hsa-miR-4505 | 9.694396 | 8.344674 | 9.694396 | 7.698118 | 7.369709 | 7.467433 | -3.323574 | 0.045921909 |
| hsa-miR-4507 | 8.527994 | 7.338143 | 8.234415 | 6.75305 | 6.616997 | 6.20364 | -2.846038 | 0.063857282 |
| hsa-miR-4516 | 13.128511 | 12.192239 | 13.579186 | 11.398096 | 11.476493 | 10.317655 | -3.738772 | 0.123785296 |
| hsa-miR-451a | 10.635118 | 9.090041 | 10.433914 | 7.74321 | 9.694396 | 8.632122 | -2.572399 | 0.317726778 |
| hsa-miR-4530 | 10.801181 | 9.962742 | 11.284945 | 9.162556 | 8.903215 | 9.398339 | -2.884363 | 0.024760447 |
| hsa-miR-4532 | 7.595952 | 6.616997 | 6.815317 | 4.46582 | 4.528973 | 4.733905 | -5.40086 | 0.019901154 |
| hsa-miR-455-3p | 4.630615 | 4.69126 | 4.571186 | 8.701416 | 6.75305 | 6.017615 | 5.761083 | 0.08589364 |
| hsa-miR-4668-5p | 3.718493 | 1.9192 | 2.280774 | 4.13036 | 4.352069 | 3.497679 | 2.555987 | 0.147661949 |
| hsa-miR-4672 | 8.374865 | 7.617967 | 7.195116 | 6.158596 | 6.017615 | 5.741398 | -3.379428 | 0.017227331 |
| hsa-miR-4687-3p | 10.593723 | 9.849133 | 11.042131 | 9.398339 | 7.675637 | 9.019009 | -3.475774 | 0.027451711 |
| hsa-miR-4734 | 5.888627 | 4.668421 | 5.510864 | 2.972188 | 3.497679 | 2.280774 | -5.422997 | 0.062575307 |
| hsa-miR-4741 | 9.443075 | 8.008252 | 9.890922 | 9.046876 | 5.697637 | 8.164983 | -2.784821 | 0.120879005 |
| hsa-miR-4787-5p | 9.090041 | 7.717032 | 8.801254 | 7.445617 | 6.658416 | 6.597104 | -3.107446 | 0.038530656 |
| hsa-miR-484 | 3.100095 | 3.54872 | 3.237845 | 4.985888 | 5.916385 | 5.228466 | 4.23206 | 0.004905513 |
| hsa-miR-487b | 2.792112 | 3.685683 | 3.943137 | 5.855749 | 3.54872 | 5.697637 | 2.949338 | 0.235044095 |
| hsa-miR-5001-5p | 8.870208 | 7.394503 | 8.19271 | 6.815317 | 5.888627 | 5.97614 | -3.799419 | 0.012244438 |
| hsa-miR-505-3p | 3.593022 | 4.400746 | 4.155607 | 7.15235 | 4.549473 | 4.489171 | 2.54419 | 0.347842616 |
| hsa-miR-572 | 9.162556 | 7.284793 | 9.443075 | 6.551124 | 3.295114 | 5.35573 | -11.817218 | 0.017422985 |
| hsa-miR-575 | 9.633341 | 8.19271 | 10.317655 | 7.595952 | 5.086088 | 8.119495 | -5.454286 | 0.018000467 |
| hsa-miR-5787 | 9.732171 | 8.937113 | 10.546623 | 9.767683 | 6.703109 | 9.345438 | -2.193486 | 0.226239156 |
| hsa-miR-6068 | 11.210763 | 9.54856 | 11.79004 | 8.714371 | 5.936221 | 7.698118 | -10.557656 | 0.018779513 |
| hsa-miR-6075 | 5.97614 | 4.352069 | 5.607302 | 3.593022 | 2.792112 | 2.892793 | -4.656335 | 0.023094327 |
| hsa-miR-6087 | 10.893592 | 10.199535 | 11.210763 | 10.023183 | 9.308811 | 9.890922 | -2.03777 | 0.019759216 |
| hsa-miR-6089 | 14.142637 | 12.927548 | 14.142637 | 12.927548 | 11.562278 | 11.672532 | -3.212036 | 0.051051302 |
| hsa-miR-6090 | 12.192239 | 11.284945 | 12.192239 | 10.893592 | 10.801181 | 10.546623 | -2.207902 | 0.080057947 |
| hsa-miR-6125 | 11.672532 | 10.945114 | 12.069148 | 10.483344 | 9.512305 | 9.923926 | -3.00856 | 0.031070271 |
| hsa-miR-638 | 11.121826 | 9.802901 | 11.562278 | 8.772935 | 7.15235 | 8.26591 | -6.798754 | 0.010059606 |
| hsa-miR-6510-5p | 7.103316 | 6.258087 | 7.94091 | 5.783729 | 3.188537 | 5.681177 | -4.64697 | 0.048309831 |
| hsa-miR-6723-5p | 5.835648 | 5.126295 | 5.835648 | 3.651221 | 5.126295 | 4.316308 | -2.353143 | 0.196357554 |
| hsa-miR-6724-5p | 8.291452 | 7.127194 | 8.462317 | 6.774293 | 6.839713 | 6.131235 | -2.600112 | 0.146049076 |
| hsa-miR-93-5p | 5.470725 | 6.720936 | 6.182175 | 8.833943 | 9.198185 | 7.595952 | 5.344598 | 0.050253156 |
| hsa-miR-98-5p | 4.985888 | 5.8073 | 5.697637 | 7.103316 | 7.302799 | 6.492344 | 2.768706 | 0.061456009 |
| hsa-miR-99a-5p | 10.023183 | 11.121826 | 10.635118 | 7.617967 | 5.835648 | 8.993941 | -8.638958 | 0.107193032 |

**Supplementary Table 1 – miRNA microarray expression data**

The miRNA expression of TNBC and adjacent normal was analyzed by miRNA microarray data. The expression of miRNA was normalized miRNA probe signal intensity by quantile method.

| **Name** | **Primer sequence** | |
| --- | --- | --- |
| Human CHK1 | forward | 5'-CTGAAGAAGCAGTCGCAGTG-3' |
|  | reverse | 5'-TTCCACAGGACCAAACATCA-3' |
| Human 18S rRNA | forward | 5'-GTCGGCGTCCCCCAACTTCTT-3' |
|  | reverse | 5'-CGTGCAGCCCCGGACATCTA-3' |

**Supplementary Table 2 – qRT-PCR primer sequence for each gene**

The sequence of qRT-PCR primers of CHK1 and 18s rRNA were described.

| **Name** | **Primer sequence** | |
| --- | --- | --- |
| Human CHK1_WT1 | forward | 5'-CTAGTTGTTTAAACGAGCTCGACATGAGTTTTCCAGCTTT-3' |
|  | reverse | 5'-CGACTCTAGACTCGAGTAAGTACAAATCTTGGGGATA-3' |
| Human CHK1_MT1 | forward | 5'-CTAGTTGTTTAAACGAGCTCGACATGAGTTTTCCCAATTTT-3' |
|  | reverse | 5'-CGACTCTAGACTCGAGTAAGTACAAATCTTGGGGATA-3' |
| Human CHK1_WT2 | forward | 5'-CTAGTTGTTTAAACGAGCTCCTTGACCAAAAACAGCTTTTG-3' |
|  | reverse | 5'-CGACTCTAGACTCGAGAAATAAATGTCAGAGCTAATCC-3' |
| Human CHK1_MT2 | forward | 5'-CTAGTTGTTTAAACGAGCTCCTTGACCAAAAACCAATTTTG-3' |
|  | reverse | 5'-CGACTCTAGACTCGAGAAATAAATGTCAGAGCTAATCC-3' |

**Supplementary Table 3 – The sequence of primer for amplify the Chk1 3’UTR gene using luciferase assay**

The prime sequence for amplifying the CHK1 3’UTR wild type (WT) and CHK1 3’UTR mutant type (MT) gene that were used in luciferase assay.


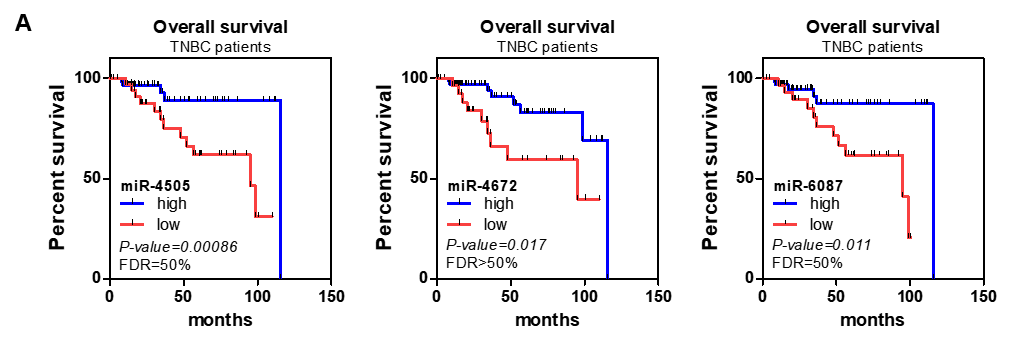


**Supplementary Figure 1– The overall survival analysis of miR-4505, miR-4672 and miR-6087 in triple-negative breast cancer.**

**(A)** Kaplan-Meier analysis of overall survival of miR-4505, miR-4672 and miR-6087 in triple-negative breast cancer patients from the KM plotter database. P-value was calculated with the log-rank test. FDR means false discovery rates. Patients were stratified into ‘low (red)’ and ‘high (blue)’ each miRNAs expression based on autoselect best cut off. Patients were stratified into ‘low (blue)’ and ‘high (red)’ miR-320c expression based on autoselect best cut off.

**
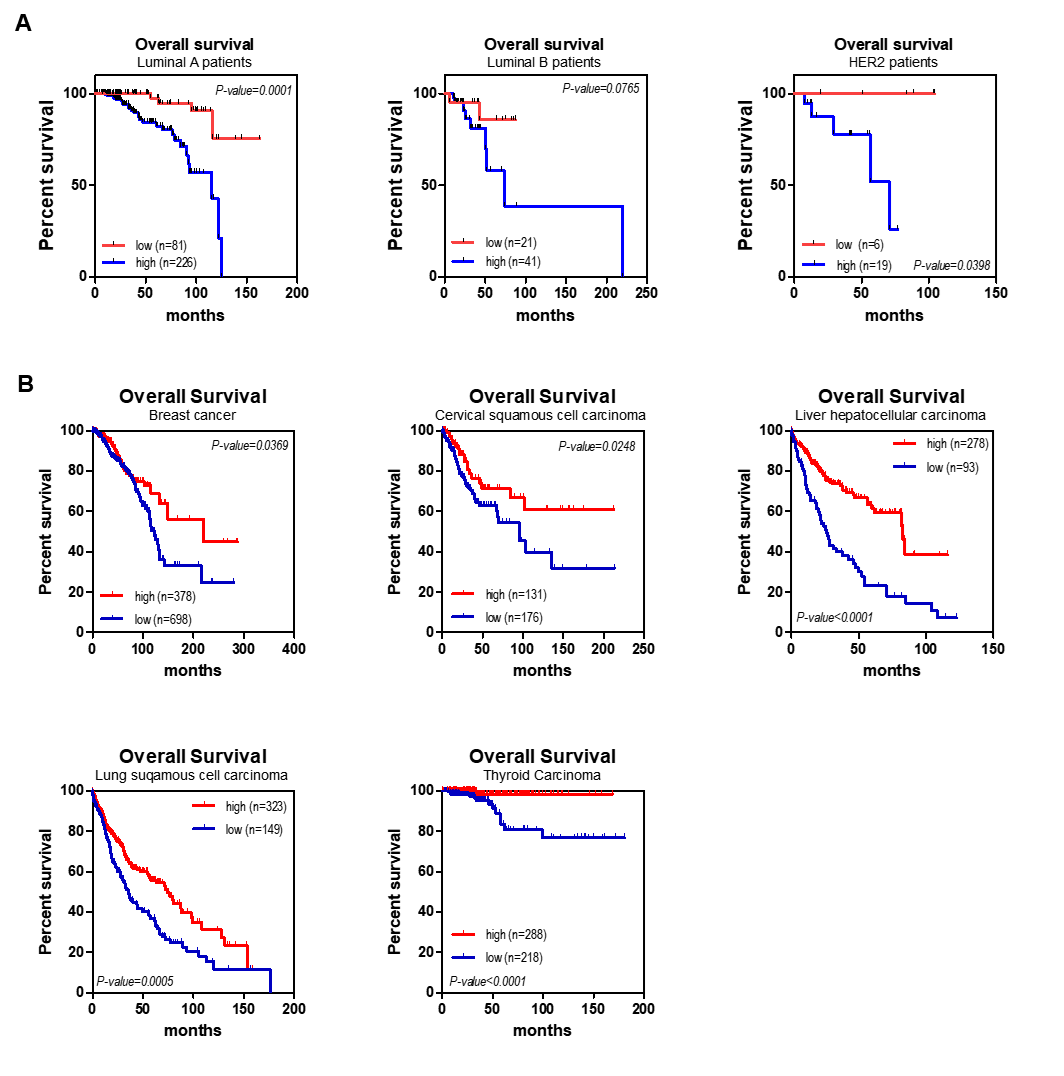
**

**Supplementary Figure 2 – Downregulated miR-320c expression is associated with poor overall survival.**

**(A)** Kaplan-Meier analysis[^3^](#_ENREF_3) of overall survival of Luminal A, Luminal B, and HER2 breast cancer patients from the KM plotter database. P-value was calculated with the log-rank test. Patients were stratified into ‘low (blue)’ and ‘high (red)’ miR-320c expression based on autoselect best cut off. (**B)** Kaplan-Meier analysis[^4^](#_ENREF_4) of overall survival of cancer patients from the KM plotter database. P-value was calculated with the log-rank test. Patients were stratified into ‘low (blue)’ and ‘high (red)’ miR-320c expression based on autoselect best cut off.

**
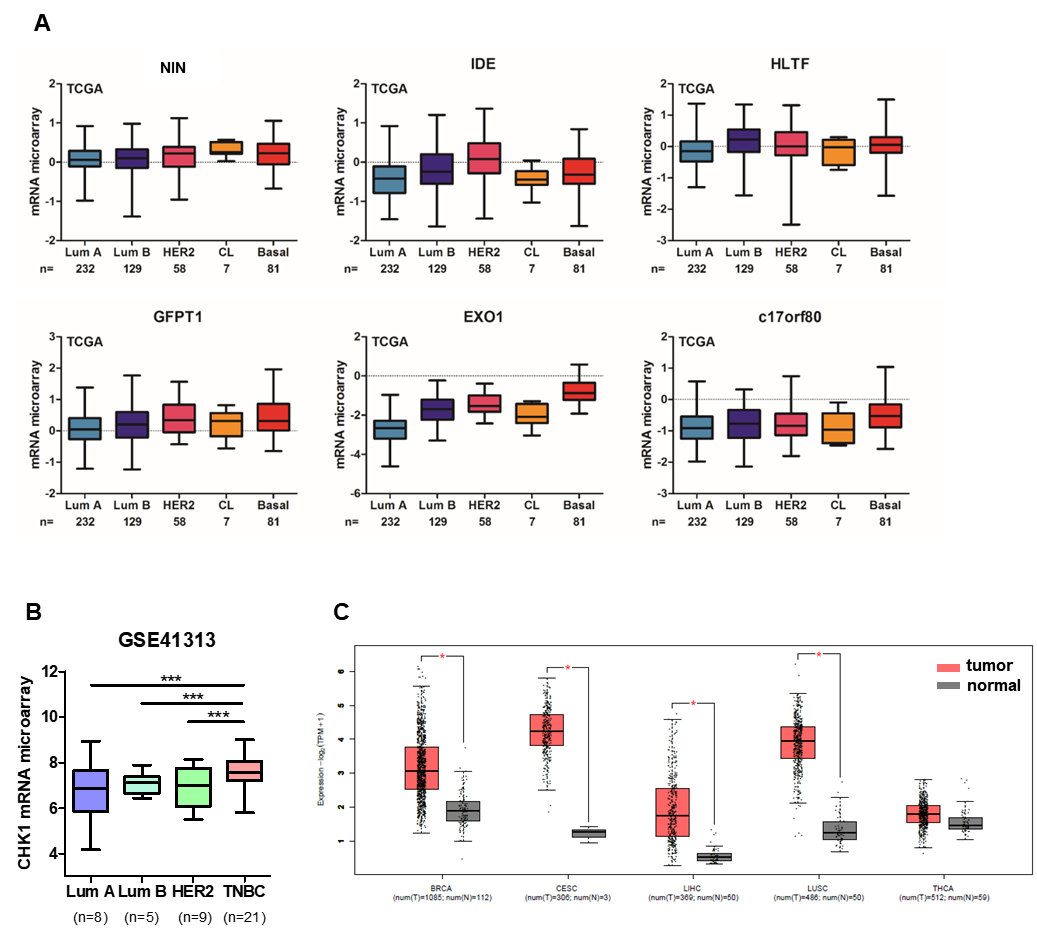
**

**Supplementary Figure 3 – CHK1 expression in various cancer.**

(A) Chk1 mRNA expression among human breast cancer subtypes. Transcriptome datasets of TCGA were analyzed using cBioPortal [^1^](#_ENREF_1)^,^ [^2^](#_ENREF_2). Lum A; Luminal A, Lum B; Luminal B, HER2, CL; Claudin-low, Basal; like TNBC. (B) GSE data showed Chk1 mRNA level in breast cancer cell lines. The values of the data mean that the mRNA expression level. Lum A; Luminal A, Lum B; Luminal B, HER2, and TNBC. (C) Chk1 mRNA expression among cancer p. Transcriptome datasets of TCGA were analyzed using GEPIA2[^5^](#_ENREF_5). BRCA; Breast cancer, CESC; Cervical squamous cell carcinoma and endocervical carcinoma, LIHC; Liver hepatocellular carcinoma, LUSC; Lung squamous cell carcinoma, THCA; Thyroid carcinoma. *; p<0.05, ***; p<0.001

**
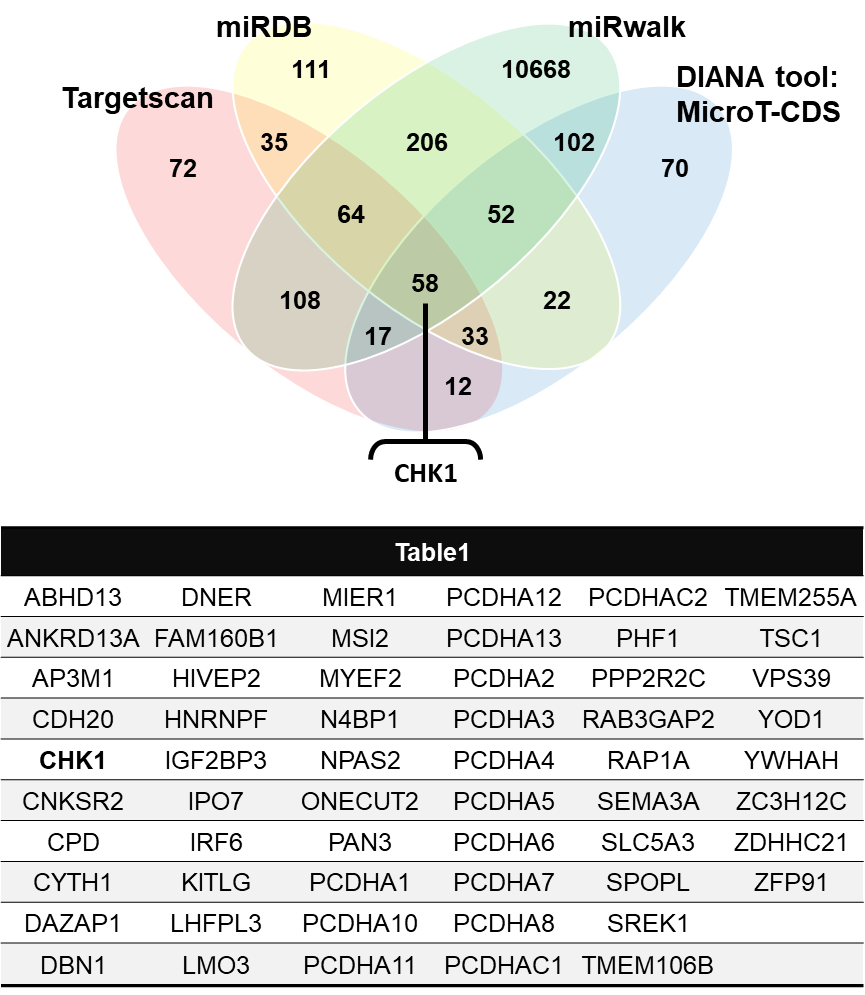
**

**Supplementary figure 4– CHK1 is a candidate of miR-320c target by using prediction DB.**

Venn diagram illustrating common gene candidates in four miRNA target prediction database. CHK1 is also contained in the common gene list of four miRNA target gene prediction database: TargetScan, miRwalk 3.0, DIANA-microT web server v5.0, and miRDB.


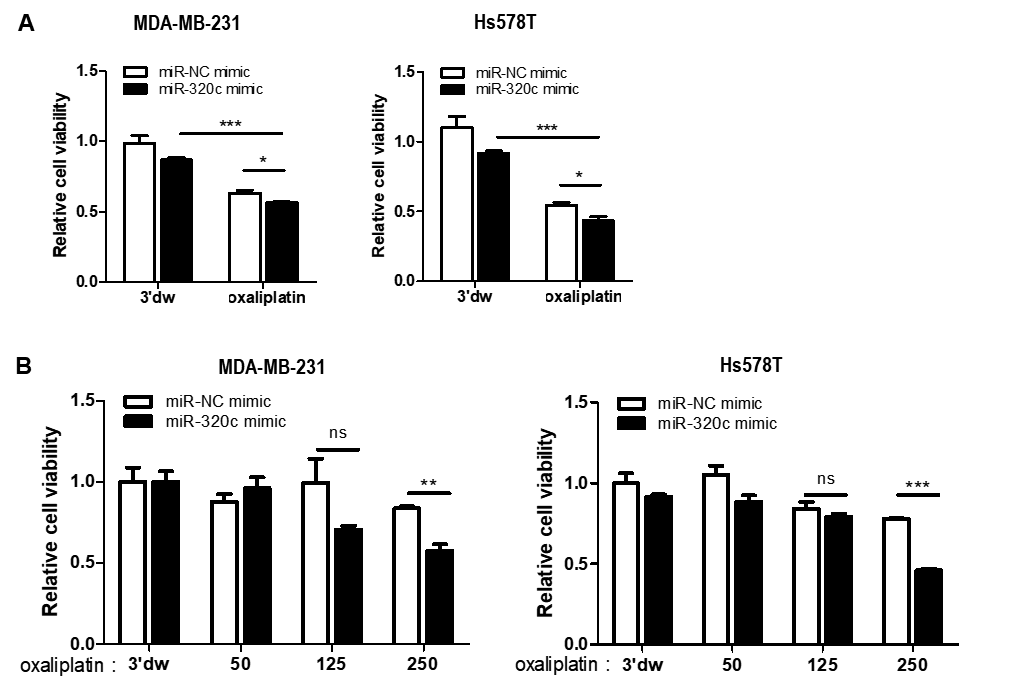


**Supplementary Figure 5 – Relative cell viability treating with oxaliplatin in TNBC cells.**

**(A)** The cell viability was measured in TNBC cells transfected with miR-320c mimic or miR-NC mimic (negative control) and treated with oxaliplatin (50μM) or 3’dw. (Left: MDA-MB-231, right: Hs578T) **(B)** Different dose of oxaliplatin oxaliplatin (50, 125, 250μM) was incubated with miR-320c mimic or miR-NC(negative control) for 1 hr and then changed completed media to give DNA repair time in TNBC cells (left : MDA-MB-231, right : Hs578T). After that, the cell viability was detected. Data were representatively shown as mean ± SD in each three independent experiments. 3’dw; 3rd deionized water, n.s; non-significant, *; p<0.05, **; p<0.01, ***; p<0.001


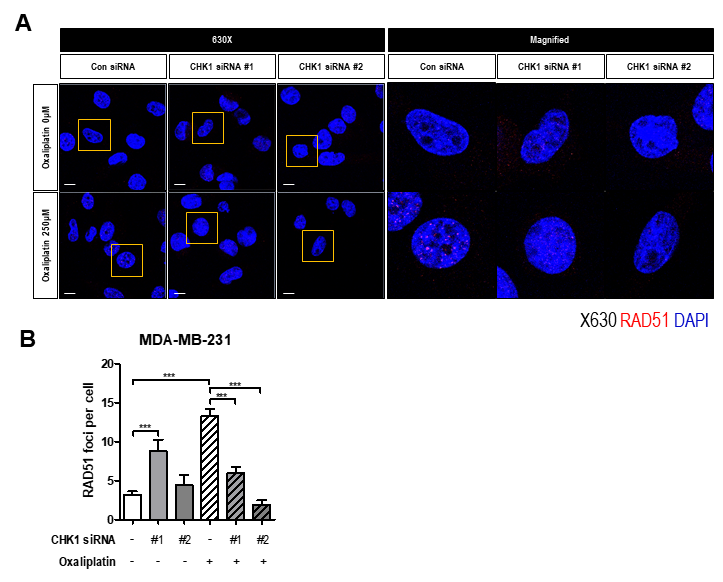


**Supplementary Figure 6 – Apoptosis and DNA damage repair was regulated by Chk1 inhibition in TNBC cells.**

**(A)** RAD51 foci formation in MDA-MB-231 cells which were transfected with Chk1 siRNA. Representative images was shown. MDA-MB-231 cells were transfected with control siRNA or Chk1 siRNA and treated with 3'DW or oxaliplatin. Each samples are stained for nuclei (DAPI; blue) and RAD51 (red). Scale bar = 10 μm. **(B)** The foci numbers per each cells were shown. Data were representatively shown as mean ± SD of at least 100 cells in each four independent experiments. ***; p<0.001


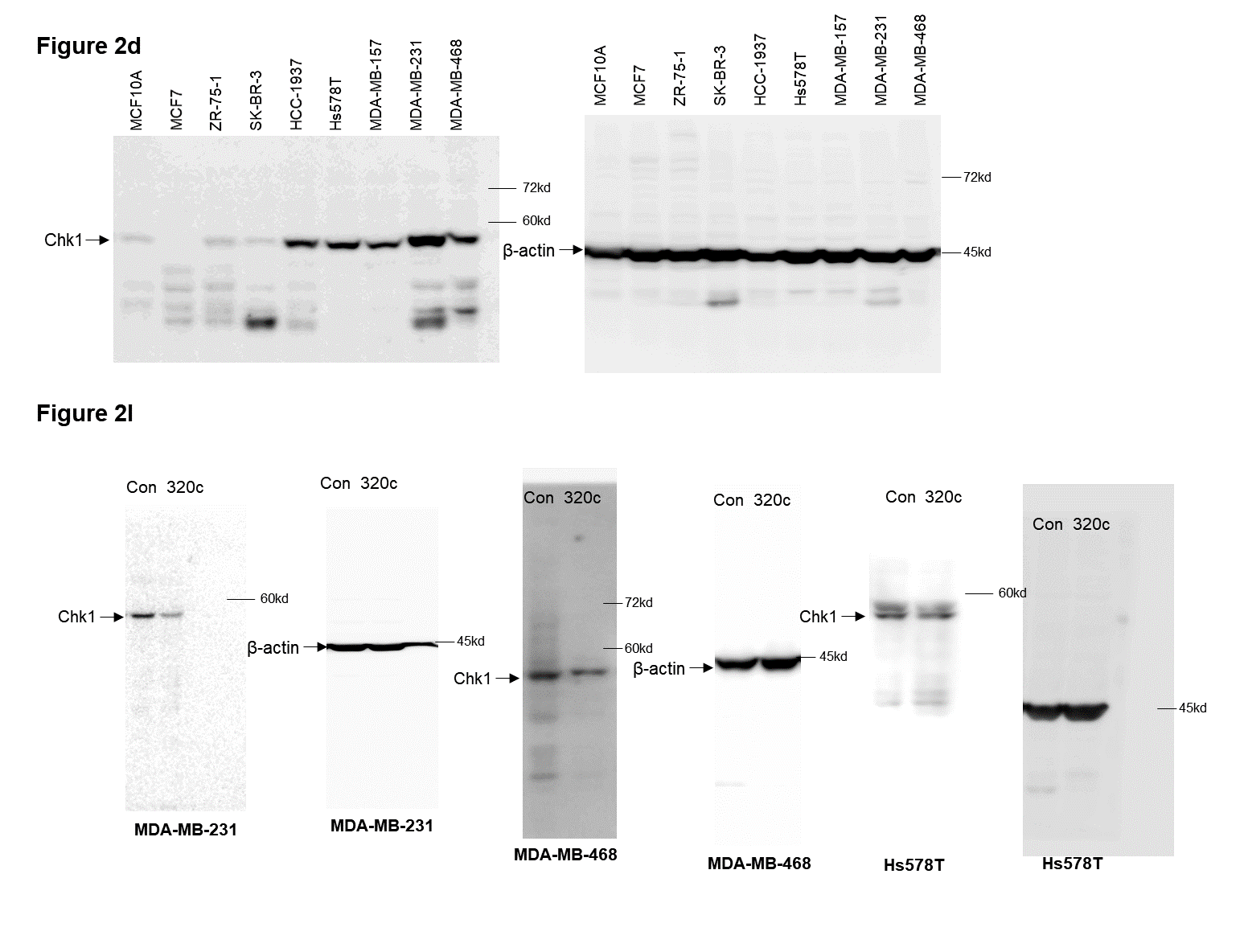

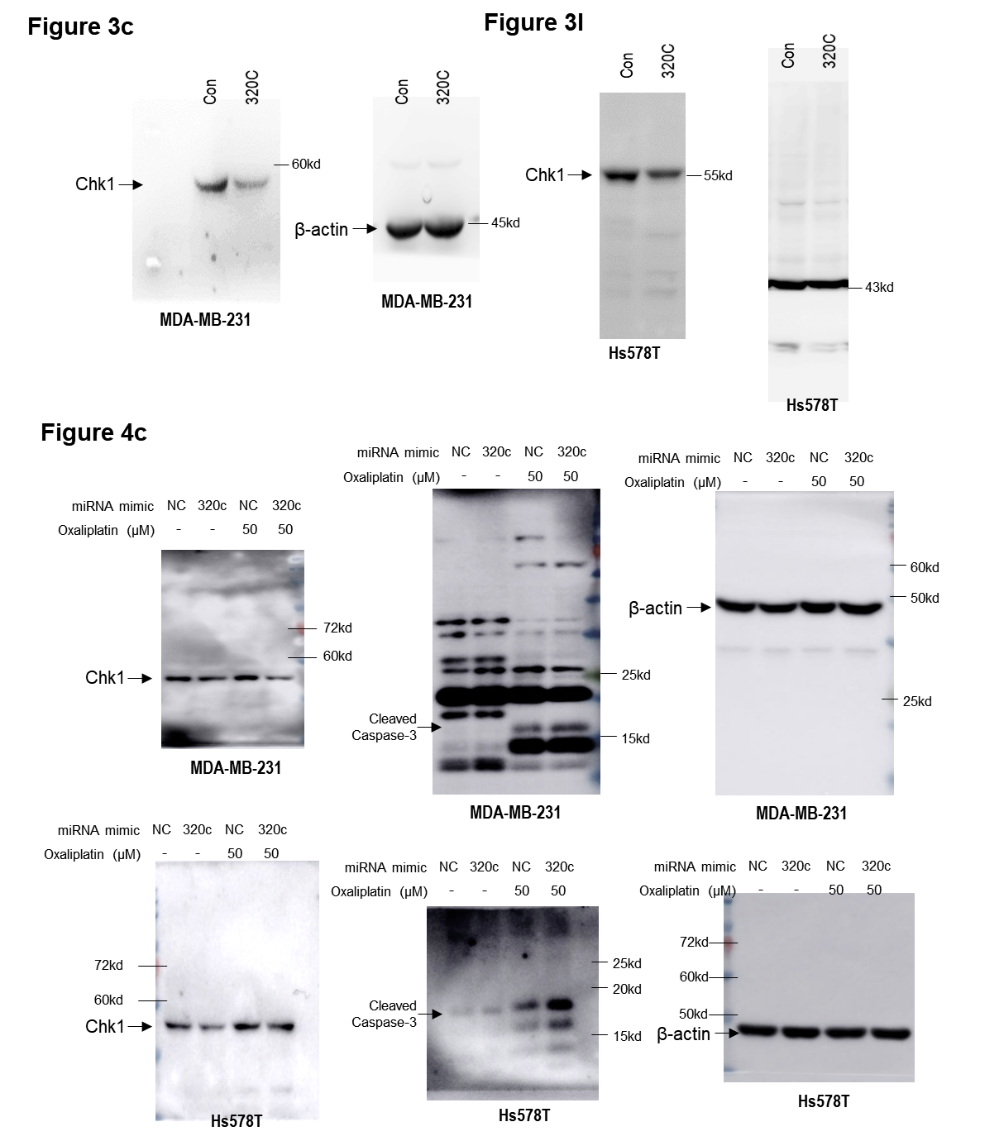


**Supplementary Figure 7 – Original data of western blot**

**References**

1 Cerami E *et al* (2012). The cBio cancer genomics portal: an open platform for exploring multidimensional cancer genomics data. *Cancer discovery* **2:** 401-404.

2 Gao J *et al* (2013). Integrative analysis of complex cancer genomics and clinical profiles using the cBioPortal. *Science signaling* **6:** pl1.

3 Lanczky A *et al* (2016). miRpower: a web-tool to validate survival-associated miRNAs utilizing expression data from 2178 breast cancer patients. *Breast cancer research and treatment* **160:** 439-446.

4 Nagy A, Lanczky A, Menyhart O, Gyorffy B (2018). Validation of miRNA prognostic power in hepatocellular carcinoma using expression data of independent datasets. *Scientific reports* **8:** 9227.

5 Tang Z, Kang B, Li C, Chen T, Zhang Z (2019). GEPIA2: an enhanced web server for large-scale expression profiling and interactive analysis. *Nucleic acids research* **47:** W556-W560.
