# Supplementary material for: Inhibition of Chk1 by miR-320c increases oxaliplatin responsiveness in triple-negative breast cancer
Source: Oncogenesis. 2020 Oct 11;9(10):91. doi: 10.1038/s41389-020-00275-x (PMC7548284; doi:10.1038/s41389-020-00275-x)
Supplement: Supplementary file 2 — Supplementary Materials and methods [file 41389_2020_275_MOESM2_ESM.docx]

**Supplementary Materials and Methods**

**Immunohistochemistry and fluorescence in situ hybridization for categorization of TNBC of breast cancer tissues**

The methods for immunohistochemistry (IHC) of breast cancer patient tissue at Seoul National University Hospital were previously described[^1^](#_ENREF_1). Briefly, Formalin-fixed, paraffin-embedded tissue sections were stained using a Benchmark automatic immunostaining device (Ventana Medical System, Tucson, AZ, USA). The samples were incubated with antibodies to estrogen receptors (ER, 1:100, 1D5; Novocastra Laboratories, Newcastle, UK), progesterone receptors (PgR, 1:200, PgR636; DAKO, Glostrup, Denmark), and HER2 (Ventana, Tucson, AZ, USA). IHC staining for ER and PgR expression was counted and categorized as positive when ≥1 % of the tumor cells were stained according to the 2010 ASCO/CAP guidelines[^2^](#_ENREF_2). Immunohistochemical expression of HER2 was assessed based on 2013 ASCO/CAP guidelines[^3^](#_ENREF_3). Presence of HER2 amplification was assessed with an additional fluorescence in situ hybridization (FISH) assay using PathVysion assay (Abbott Molecular, Downers Grove, IL, USA) in those cases with equivocal HER2 protein expression (IHC 2+) on IHC. Interpretation for HER2 gene amplification by FISH assay was confirmed based on the ≥2.0 ratio of HER2 gene copy number to chromosome 17 copy number, or ≥6.0 average HER2 signal per each tumor cell as defined by the 2013 ASCO/CAP guidelines[^3^](#_ENREF_3). Triple-negative breast cancer was defined when ER, PgR, and HER2 were negative in IHCs and HER2 FISH.

**Transfection of miRNA mimics and miRNA vector**

As miRNA, miRNA mimics were used. Thus, miRVana^TM^ miR-320c mimic (Ambion, Austin, TX, USA) was reverse-transfected at a final concentration of 30 nM using the siPORT^TM^ NeoFXTM transfection agent (Ambion) according to the manufacturer’s instructions. Control experiments were transfected with a miRVana^TM^ miR-negative control mimic (Negative control #1, Ambion). For transient overexpression of miRNA expression, the miR-320c lentiviral vector and empty vector (Abm^®^, Richmond, Canada) were transfected into the cells using the FuGENE^®^ HD transfection reagent (Promega, Madison, USA) and incubated for 48 h.

**Lentiviral vector transduction**

Stable overexpression of miR-320c in MDA-MB-231 and Hs578T cells was established using miRNA lentiviral vectors. The construct to overexpress miR-320c and the corresponding negative control construct was purchased from Abm® (Richmond, Canada). For the generation of the virus, HEK293T cells were transfected with the constructs and lentiviral packaging vector (Abm®) by lentifectin (Abm®) according to the manufacturer’s protocol, and viruses were collected 48 h after the transfection. MDA-MB-231 and Hs578T cells were infected with the miR-320c overexpressing or negative control viruses by the spin-fection method and selected by puromycin (Gibco) and GFP. The miR-320c expression levels of infected cells were confirmed by miRNA expression analysis. Stable cell lines were cultured in DMEM (WelGENE) with 10% FBS (Gibco) and puromycin (2 μg/mL, from Gibco).

**Western blotting**

Protein contents were measured using the Bicinchoninic acid Solution (Sigma) and Copper (Ⅱ) sulfate solution (Sigma). Proteins were separated on SDS-PAGE gel. Western blotting was performed using standard methodology. Antibodies were diluted at 1:1000-1:2000 with 1% skimmed milk in PBS containing 0.1% Tween® 20 (Sigma). β-actin was used as a loading control.

**Quantitative RNA isolation and quantitative RT-PCR (qRT-PCR)**

We extracted total RNA including miRNA and other small RNAs from cultured cells with Trizol® (Ambion). Then, 500 ng of total RNA was reverse-transcribed with the TaqMan® MicroRNA Reverse Transcription Kit (Applied Biosystems, Foster City, CA, USA). Quantitative RT-PCR for miRNA expression analysis was performed with the Taqman® Universal PCR Master Mix (Applied Biosystems) using the LightCycler® System (Roche, Basel, Switzerland) and RNU48 was used as an internal control. For detecting gene expression level, 5 µg of total RNA was reverse-transcribed by using M-MLV Reverse Transcriptase (Promega, Madison, WI, USA), and synthesized cDNA was used for qPCR, performed using SYBR Green qPCR Master Mix (PCR Biosystem, London, UK) and LightCycler® System (Roche). The relative expression levels of the target gene or miRNA were calculated by 2^ (-∆∆Cq) method, normalized against the level of 18s rRNA or RNU48, which was used for the endogenous control.RT-PCR (qRT-PCR)

**Dual-Luciferase reporter assay**

Human Chk1 3'-UTR that included the predicted miR-320c binding sequences and Chk1 3’-UTR that include mutated miR-320c binding sequences were amplified by PCR from MDA-MB-231 cDNA. The amplified PCR fragments were cloned into pmirGLO Dual-Luciferase vector. These luciferase constructs were co-transfected in HEK293T cells with miR-320c mimic or miR-NC mimic, using Lipofectamine 2000 reagents (Invitrogen). Luciferase activities were measured using the Dual-Luciferase Reporter Assay System (Promega), according to the manufacturer’s manual.

**Immunostaining (ICC/IF/IHC) microscopy**

For immunocytochemistry (ICC), cells incubated on coverslips were fixed by 10% Neutral Buffered Formalin (Biosesang, Seongnam, Korea) for 10 min. After then, the cell was permeabilized for 15 minutes and incubated with Anti-phospho-Histone H2A.X (Ser139) Antibody (Merck Millipore, Darmstadt, Germany) or Anti-Rad51 antibody (Abcam, Cambridge, UK) at 4 ℃ overnight. For Immunofluorescence (IF), paraffin sections of xenograft tumors were used. First, the slides were rehydrated in Histoclear, 100%, 95%, 80% and 70% ethanol in order. Then antigen retrieval was proceed using TintoRetriver Pressure Cooker (Bio SB, Goleta, CA) and Borg Decloaker RTU (Biocare medical, Pacheco, CA) according to manufacturer’s manual. The slides were incubated with Chk1 antibody (Abcam) or Anti-phospho-Histone H2A.X (Ser139) Antibody (Merck Millipore) at 4 ℃ overnight. For immunofluorescence staining microscopy, DAPI staining was used for visualizing the nuclei. Images were visualized by Confocal Laser Scanning Microscope (LSM 700, Carl Zeiss, Oberkochen‎, Germany) and each image was captured with identical exposure settings.

For immunofluorescence, DAPI staining was used for visualizing the nuclei. Images were visualized by Confocal Laser Scanning Microscope (LSM 700, Carl Zeiss, Oberkochen‎, Germany) and each image was captured with identical exposure settings. The γ-H2AX and RAD51 foci formation were analyzed with i-Solution software (IMT, Gyungnam, Korea) and Chk1 intensity was analyzed with ImageJ software (NIH) and normalized to DAPI intensity to decrease the effect of slightly different focal z-planes. For immunohistochemistry, staining with cleaved caspase 3 antibody (Cell Signaling Technology, Beverly, MA, USA) was performed using Vectastatin ABC kit (Vector Laboratories, Burlingame, CA, USA) according to manufacturer’s instruction. Staining was visualized by NovaRED substrate (Vector Laboratories) and counterstaining was performed with hematoxylin. The cleaved-caspase 3 staining intensity was analyzed with i-Solution software.

**Clonogenic assay**

To determine the effectiveness of the drug, a clonogenic assay was conducted by the followed method. Plate 0.5–1 × 103/mL of cells on a 6-well plate. Then cisplatin, carboplatin, and oxaliplatin (Sigma) was treated in each concentration. The cells were incubated in a CO2 incubator at 37 ℃ for 1–3 weeks until cells in control plates had formed good size colonies (at least, 50 cells per colony). After incubation, cells were fixed and stained with 0.5% crystal violet solution (with 10% ethanol).

**Caspase-3 activation assay**

Cells which show stable overexpression of miR-320c and corresponding negative control cell were used. Plate 1×105/mL of cells on 100 cm2 plates. Then the 50 µM of oxaliplatin (Sigma) was treated to each plate the following day and the cells were incubated in a CO2 incubator at 37 ℃ for 48hrs. After incubation, cells were harvested and the subsequent procedures were carried out by ApoAlert Caspase Colorimetric Kit (Clontech Laboratories, Mountain View, CA, USA). The final samples were read at 405nm in a multi-well spectrophotometer.

**Xenograft mice and intratumoral miRNA transfection**

All animal experiments were approved by the Institutional Review Board of the Yonsei University College of Medicine and were performed in specific pathogen-free facilities according to the university’s guidelines for the Care and Use of Laboratory Animals (2018–0155). All mice were housed in pathogen-free facilities, in a 12 hours light/dark cycle in ventilated cages, with chow and water supply ad libitum. Thus, 8-week old female Balb/c-nude mice (Orient, Seongnam, Korea) were inoculated subcutaneously with 2.0 × 106 MDA-MB-231/A cells into each flank under 100 µL of saline/zoletil/rompun (7:1:1) anesthesia. When tumors reached an average volume of 100–150 mm3, after 2 weeks, the mice were then treated with intratumoral injection of synthetic miRNA and intraperitoneal (ip) injection of oxaliplatin (Tocris Bioscience, Bristol, UK) or 3’DW (3rd distilled water).. Different types of synthetic miRNA (miR-320c/miR-NC) (6.25 μg) of complexed with 1.6 μL siPORT™ NeoFX™ Transfection Agent (Ambion) in 50 μL PBS were delivered intratumorally in 3–5 day intervals for each tumor. Then, 3’DW or oxaliplatin was applied with an intraperitoneal injection (2 mg/kg) in 3 - 5 day intervals. From palpable tumor formation until termination, tumor sizes were measured every 3 to 5 days using calipers, and tumor volume was calculated with the following formula: length × width2 × 0.5. Mice were sacrificed with an overdose of anesthesia, and tumors were harvested for immunohistochemistry and other analyses.

**Reference**

1 Chung SY *et al* (2016). Oncogenic role of SIRT1 associated with tumor invasion, lymph node metastasis, and poor disease-free survival in triple negative breast cancer. *Clinical & experimental metastasis* **33:** 179-185.

2 Hammond ME *et al* (2010). American Society of Clinical Oncology/College of American Pathologists guideline recommendations for immunohistochemical testing of estrogen and progesterone receptors in breast cancer. *Archives of pathology & laboratory medicine* **134:** 907-922.

3 Wolff AC *et al* (2013). Recommendations for human epidermal growth factor receptor 2 testing in breast cancer: American Society of Clinical Oncology/College of American Pathologists clinical practice guideline update. *Journal of clinical oncology : official journal of the American Society of Clinical Oncology* **31:** 3997-4013.
